# Sexually transmitted co-infections as biological modifiers of HPV-driven cervical carcinogenesis: a systematic integrative review

**DOI:** 10.3389/fcimb.2026.1938728

**Published:** 2026-10-28

**Authors:** Emad Alqassim, Arwa A. Al-Qahtani, Ahmad Y. Alqassim, Ahmed A. Al-Qahtani

**Affiliations:** 1 Department of Anatomic Pathology, Division of Pathology and Laboratory Medicine, King Faisal Specialist Hospital and Research Center, Riyadh, Saudi Arabia; 2 Department of Pathology, College of Medicine, Alfaisal University, Riyadh, Saudi Arabia; 3 Department of Family Medicine, College of Medicine, Imam Mohammad Ibn Saud Islamic University (IMSIU), Riyadh, Saudi Arabia; 4 Department of Family and Community Medicine, College of Medicine, Jazan University, Jazan, Saudi Arabia; 5 Department of Infection and Immunity, Research Center, King Faisal Specialist Hospital and Research Center, Riyadh, Saudi Arabia; 6 Department of Microbiology and Immunology, College of Medicine, Alfaisal University, Riyadh, Saudi Arabia

**Keywords:** carcinogenesis, cervical cancer (CC), *Chlamydia trachomatis* (CT), herpes simplex virus type 2 (HSV-2), human papillomavirus (HPV), HIV, co-infection

## Abstract

Persistent high-risk human papillomavirus (HR-HPV) infection is necessary but not sufficient for cervical cancer, which caused approximately 350,000 deaths in 2022. Co-infections with *Chlamydia trachomatis* (CT), herpes simplex virus type 2 (HSV-2), and HIV may modify cervical carcinogenesis, but causal inference is constrained by confounding, study heterogeneity, and limited mechanistic validation in human tissue. This systematic integrative review (PRISMA 2020) searched PubMed, Embase, Scopus, Web of Science, and the Cochrane Library (January 2015 to March 2026); risk of bias was assessed using the Newcastle-Ottawa Scale and ROBINS-I, and certainty of evidence using GRADE. HIV co-infection shows the strongest evidence: approximately 6-fold risk elevation, a confirmed CD4 dose-response, and residual risk that persists despite antiretroviral therapy (GRADE: high). CT is associated with 2-4-fold elevations in observational studies, but substantial heterogeneity, residual sexual-behavior confounding, funnel-plot asymmetry suggesting publication bias, and attenuation of the HPV-adjusted independent effect (OR 1.76, 95% CI 1.03-3.01) leave causality uncertain (GRADE: Low-Moderate). HSV-2 associations are inconsistent: pooled cervical cancer risk was elevated (OR 3.01, 95% CI 2.24-4.04), but most estimates were unadjusted for HPV status, prospective studies are non-significant, and mechanistic evidence derives from non-human models (GRADE: low). A context-dependent syndemic effect appears in HIV co-infected populations. In conclusion, HIV meets criteria for a probable causal modifier of HPV-driven carcinogenesis, supporting WHO-endorsed intensified screening for women living with HIV; CT’s role remains uncertain pending rigorous confounder control; and HSV-2 does not meet the threshold for independent co-factor classification. Priorities include prospective cohorts and human cervical-tissue mechanistic validation.

## 1 Introduction

### 1.1 Global burden of cervical cancer

Cervical cancer affects approximately 660,000 women annually, with an estimated 350,000 deaths in 2022 (GLOBOCAN), making it the fourth most common cancer in women worldwide and the leading cause of cancer-related death in 23 low- and middle-income countries (LMICs) (Bray et al., 2024). Approximately 94% of deaths occur in LMICs, where vaccine coverage, screening access, and treatment availability remain inadequate (World Health Organization, 2023a). Age-standardized incidence in Eastern Africa exceeds 40 per 100,000, compared with fewer than 5 per 100,000 in Western Europe (Sung et al., 2021). Earlier GLOBOCAN data estimated 528,000 new cases in 2012, illustrating the persistent pre-elimination burden (Torre et al., 2015). Temporal trend analyses project increasing incidence in several high-burden LMICs absent accelerated intervention (Shao et al., 2026). Modeling analyses demonstrate that achieving the WHO 90–70–90 targets would avert up to 13.4 million cervical cancer cases by 2069 across 78 LMICs (Brisson et al., 2020). Global first-dose HPV vaccine coverage stands at approximately 27%, well below the elimination target (Han et al., 2025). HIV co-infection carries the single largest relative risk elevation of the three STIs considered here, detailed fully in Section 5.1.1.

### 1.2 HPV natural history and carcinogenesis

HPV comprises over 200 genotypes, of which approximately 14 are classified as high-risk by the International Agency for Research on Cancer (IARC) (Muñoz et al., 2003). HPV-16 and HPV-18 together account for approximately 70–76% of invasive cervical cancers. In approximately 90% of infected women, HPV is cleared immunologically within 12–24 months (Rodriguez et al., 2008). Persistent infection is associated with constitutive *E6* and *E7* oncoprotein expression following viral genome integration and disruption of the *E2* regulatory gene (Soheili et al., 2021; Kobayashi et al., 2024). *E6* promotes *p53* degradation and telomerase activation; *E7* inactivates the retinoblastoma protein (*pRb*) (Mirabello et al., 2017; Rosendo-Chalma et al., 2024). High conservation of *E7* sequences across HPV-16 strains is directly linked to oncogenic potency (Mirabello et al., 2017; Rosendo-Chalma et al., 2024). These molecular events underlie sustained proliferation, apoptosis resistance, and genomic instability over a typically 10–20-year progression interval (Schiffman et al., 2016). The local cervicovaginal microbiome and immune microenvironment also shape this trajectory, a theme returned to in Section 7.2.

### 1.3 The polymicrobial context of STIs

STIs co-occur frequently owing to shared behavioral risk factors. The WHO estimates 374 million new curable STI infections annually (World Health Organization, 2023b). CT accounts for approximately 128 million new infections per year; HSV-2 affects an estimated 13.2% of persons aged 15–49 globally (James et al., 2020); and HIV affects approximately 39 million people worldwide (UNAIDS, 2023). In the Middle East and Gulf region, reported STI prevalence probably underestimates true burden owing to ascertainment bias from stigma, limited surveillance, and restricted care-seeking (Akbari et al., 2023).

### 1.4 Rationale and objectives

Despite the recognized association between STI co-infection and cervical cancer outcomes, no methodologically rigorous integrative synthesis simultaneously addresses epidemiological magnitude, heterogeneity (including null findings), mechanistic plausibility with explicit evidence-level characterization, and clinically actionable guidance linked to evidence strength. This review addresses that gap with four objectives: (1) to synthesize epidemiological evidence on STI co-infection associations with cervical cancer outcomes, incorporating heterogeneity and null findings; (2) to critically evaluate biological mechanisms with explicit characterization of evidence level; (3) to identify inconsistencies and under-represented populations; and (4) to provide recommendations explicitly linked to current guideline endorsement and evidence tier.

## 2 Materials and methods

### 2.1 Protocol, registration, and reporting standards

This systematic integrative review was conducted in accordance with PRISMA 2020 reporting guidelines (Page et al., 2021) and the integrative review methodology of Whittemore and Knafl (2005). The review protocol, including the pre-specified search strategy, inclusion criteria, and analysis methods, was developed in advance and followed consistently throughout the review. The protocol was not formally registered on PROSPERO or an equivalent registry; this is noted as a limitation in Section 10.

### 2.2 Search strategy

#### 2.2.1 Databases, date range, and language restriction

Five databases were systematically searched: PubMed/MEDLINE, Embase, Scopus, Web of Science, and the Cochrane Library. Supplementary grey literature sources included WHO reports, IARC monographs, GLOBOCAN 2022, and CDC/WHO STI and HIV guidelines. The last search was conducted in March 2026. The primary date range was January 2015 to March 2026; seminal pre-2015 studies were selectively included. The search was restricted to English-language publications, which may systematically under-represent data from high-burden regions where relevant research is published in other languages. The direction and magnitude of this potential bias cannot be determined.

#### 2.2.2 Boolean search strings

HPV–CT: (“Human papillomavirus” OR “HPV” OR “cervical cancer” OR “CIN”) AND (“*Chlamydia trachomatis*” OR “chlamydial infection”) AND (“co-infection” OR “coinfection” OR “risk factor” OR “cervical carcinogenesis”)
HPV–HSV-2: (“Human papillomavirus” OR “HPV” OR “cervical cancer” OR “CIN”) AND (“herpes simplex virus 2” OR “HSV-2”) AND (“co-infection” OR “carcinogenesis” OR “risk factor”)
HPV–HIV: (“Human papillomavirus” OR “HPV” OR “cervical cancer” OR “CIN”) AND (“HIV” OR “human immunodeficiency virus” OR “women living with HIV”) AND (“co-infection” OR “cervical carcinogenesis” OR “antiretroviral” OR “CD4” OR “immunosuppression”)
MeSH terms applied: Papillomaviridae; Uterine Cervical Neoplasms; *Chlamydia trachomatis*; Simplexvirus; HIV Infections; Coinfection; Cervical Intraepithelial Neoplasia; Carcinogenesis; Immune Evasion; Cytokines.

The conceptual search logic above was translated into the native syntax of each database searched, using identical concepts, Boolean structure, and filters (Humans; English language; publication date 2015/01–2026/03) throughout. Full strings and yields are given below.

#### 2.2.3 Database-specific search strings

##### 2.2.3.1 PubMed/MEDLINE (native interface; searched directly)

HPV–CT: (“Human papillomavirus”[tiab] OR “HPV”[tiab] OR “cervical cancer”[tiab] OR “CIN”[tiab]) AND (“*Chlamydia trachomatis*”[tiab] OR “chlamydial infection”[tiab]) AND (“co-infection”[tiab] OR “coinfection”[tiab] OR “risk factor”[tiab] OR “cervical carcinogenesis”[tiab]), 204 records. HPV–HSV-2: (“Human papillomavirus”[tiab] OR “HPV”[tiab] OR “cervical cancer”[tiab] OR “CIN”[tiab]) AND (“herpes simplex virus 2”[tiab] OR “HSV-2”[tiab]) AND (“co-infection”[tiab] OR “carcinogenesis”[tiab] OR “risk factor”[tiab]), 64 records. HPV–HIV: (“Human papillomavirus”[tiab] OR “HPV”[tiab] OR “cervical cancer”[tiab] OR “CIN”[tiab]) AND (“HIV”[tiab] OR “human immunodeficiency virus”[tiab] OR “women living with HIV”[tiab]) AND (“co-infection”[tiab] OR “cervical carcinogenesis”[tiab] OR “antiretroviral”[tiab] OR “CD4”[tiab] OR “immunosuppression”[tiab]), 1,950 records.

##### 2.2.3.2 Embase, Scopus, Web of Science, Cochrane Library

The same conceptual logic was translated into each platform’s native syntax (Embase:.mp./Emtree; Scopus: TITLE-ABS-KEY(); Web of Science: TS=(); Cochrane::ti,ab,kw). PubMed/MEDLINE returned 2,218 records across the three strings (204, 64, and 1,950 for CT, HSV-2, and HIV, respectively), and the four remaining databases returned 629 records combined, for a total of 2,847 records identified. Because most records from these four databases had already been retrieved by PubMed/MEDLINE, they contributed few unique records after the 743 duplicates were removed. The full cascade is reported in Section 2.4 and **Figure 1**.

**Figure 1:**
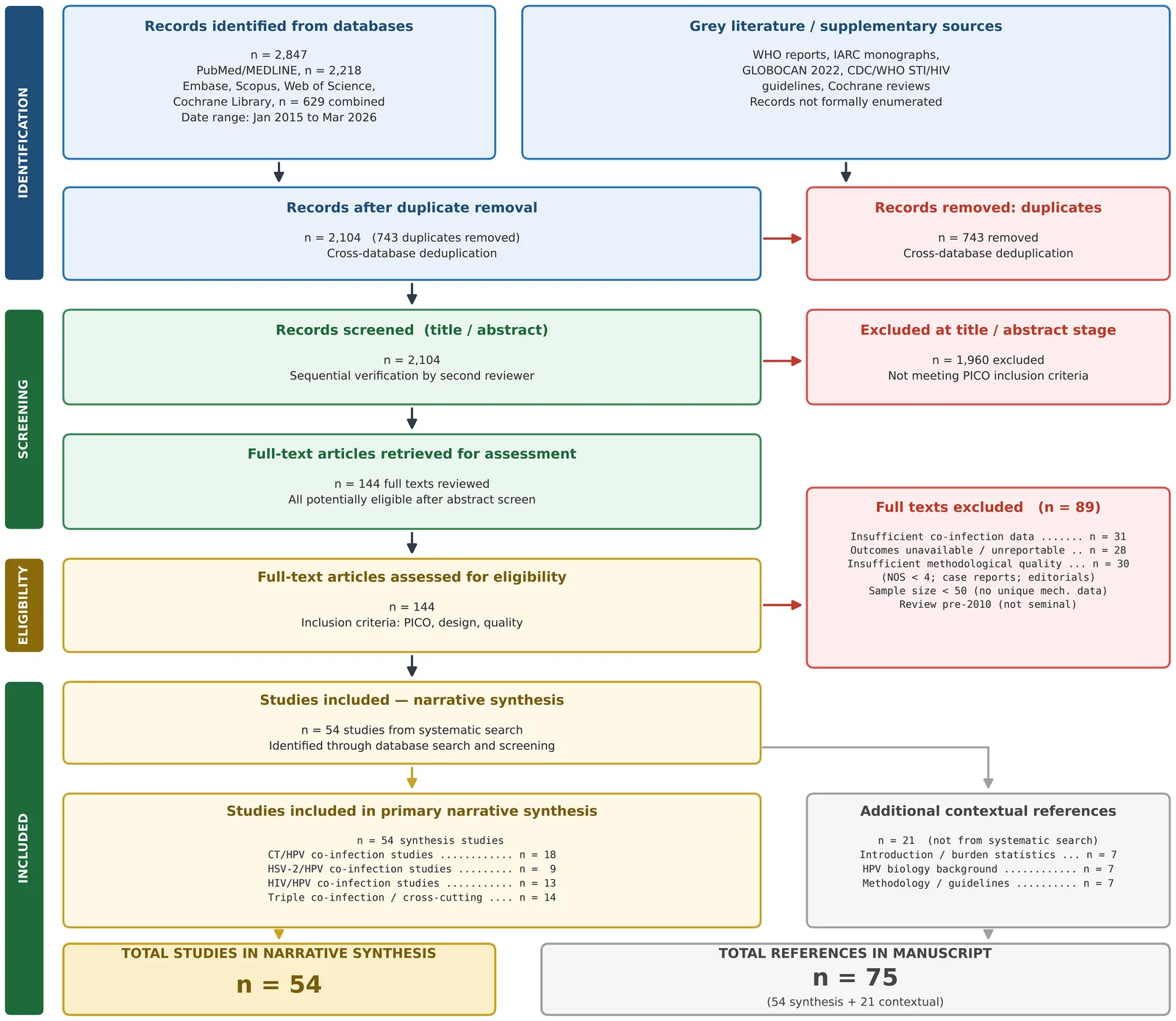
PRISMA 2020 flow diagram documenting the identification, screening, eligibility assessment, and inclusion of studies for this systematic integrative review (flow counts shown in diagram).

### 2.3 Eligibility criteria

#### 2.3.1 Inclusion criteria (PICO-structured)

Population: Women with confirmed HPV infection or at cervical cancer risk; all ages; all geographic settings. Exposure: Co-infection with CT, HSV-2, HIV, or multiple STIs concurrent with HPV. Comparator: HPV mono-infection; HIV-negative women; women without the specified STI. Outcomes: HPV persistence; CIN grades 1–3; invasive cervical cancer; molecular endpoints including cytokine profiles, viral load, and biomarker expression. Study designs: Cohort, case–control, cross-sectional, systematic reviews and meta-analyses, and mechanistic *in vitro*/*in vivo* studies.

#### 2.3.2 Exclusion criteria

Studies exclusively examining non-cervical HPV disease; observational studies with sample sizes below 50 (unless providing unique mechanistic data); case reports and editorials without primary data; and reviews published before 2010 unless of seminal historical importance.

### 2.4 Study selection and data extraction

The primary reviewer conducted systematic screening of titles, abstracts, and full-text articles. All inclusion decisions, borderline exclusions, and extracted data were independently verified by a second author, with disagreements resolved by consensus. This sequential verification protocol, although not equivalent to fully independent concurrent dual screening (the PRISMA 2020 standard), substantially reduces selection bias. Inter-rater reliability statistics were not calculated given the sequential design.

PRISMA flow summary: 2,847 records were identified across the five databases (PubMed/MEDLINE, n = 2,218; Embase, Scopus, Web of Science, and Cochrane Library, n = 629 combined)); 743 duplicates were removed; 2,104 records were screened; 1,960 were excluded at the abstract stage; 144 full texts were assessed for eligibility; and 89 were excluded (insufficient co-infection data, n = 31; outcomes unavailable, n = 28; insufficient quality, n = 30). In total, 54 studies were included in the narrative synthesis (**Figure 1**): 18 on CT-HPV co-infection, 9 on HSV-2–HPV, 13 on HIV–HPV, and 14 on triple co-infection or cross-cutting themes. The 54 included sources refer to studies identified through the systematic search and eligibility process; an additional 21 references cited for background context, methodological frameworks, and clinical guidelines are not included in this count (75 total references).

### 2.5 Data extraction

Standardized data extraction captured: author, year, country, study design, sample size, demographics, STI confirmation method (serological versus NAAT), HPV genotyping methodology, effect estimates (OR, HR, RR with 95% CI), follow-up duration, confounders adjusted, and quality score. Mechanistic studies additionally documented experimental model, cell line or species, intervention, replication evidence, and molecular endpoints. Heterogeneity statistics (I² where reported) were recorded.

### 2.6 Risk of bias and mechanistic study assessment

Observational studies were assessed using the Newcastle–Ottawa Scale (NOS; 9-point: scores 7–9, high quality; 4–6, moderate; below 4, low). ROBINS-I was applied to non-randomized cohort designs. The GRADE framework was applied to all key epidemiological claims; a full evidence profile is presented in **Table 1**. Risk of bias was evaluated at the domain level across four dimensions: (1) selection bias, including the appropriateness of case and control source populations and non-participation bias in case–control designs; (2) exposure assessment bias, including serological versus NAAT-based STI detection and the misclassification implications of each; (3) confounding, including the completeness of sexual behavior covariate adjustment and the use of objective versus self-reported confounder measurement; and (4) outcome bias, including the consistency of cervical outcome ascertainment across study populations. Studies scoring below 5 on NOS are designated high risk of bias; studies with NOS 5–6 are moderate risk; NOS 7–9 are low risk. High-risk studies include the Ferrera et al. (2023) and Ngombe Mouabata et al. (2024) cross-sectional datasets, which provide important geographic representation but have limited adjustment for confounding. Sensitivity interpretation excluding these high-risk studies does not materially alter the primary conclusions for CT: the association with CIN and cervical cancer risk persists in higher-quality prospective and pooled case–control designs, though the effect size is attenuated. For HSV-2, the available studies consist predominantly of case–control designs (moderate-to-high bias risk arising from exposure and selection domains), and exclusion of these would leave an evidence base of two higher-quality studies (Lehtinen et al., 2002; Smith et al., 2002) with discordant findings, reinforcing the GRADE: low classification.

**Table 1:** GRADE evidence profile — STI Co-infections as modifiers of HPV-driven cervical carcinogenesis.

GRADE domain	HIV–HPV	CT–HPV	HSV-2–HPV	Downgrading rationale (CT)	Downgrading rationale (HSV-2)	Final GRADE
Study design (starting point)	Observational (starts at Low; upgraded for dose–response)	Observational (starts at Low)	Observational (starts at Low)	Case–control dominant; cross-sectional designs preclude temporality	Case–control dominant; most estimates unadjusted for HPV status	—
Risk of bias	Not serious: HIV status objectively measured; CD4 dose–response across multiple independent cohorts	Serious: serology/NAAT misclassification; exposure ascertainment bias; incomplete sexual behavior adjustment	Serious: serology reflects lifetime exposure; HSV-1/HSV-2 cross-reactivity in older assays; recall bias in case–control designs	Downgrade 1 level: serious risk of bias from exposure misclassification and selection bias in dominant case–control designs	Downgrade 1 level: serious risk of bias from unadjusted HPV status and case–control dominance	—
Inconsistency	Not serious: I² 40–60% for ART outcomes (Kelly et al., 2018a); directionally consistent across all regions and designs	Serious: I² significant and substantial (Zhu et al., 2016); wide OR range across designs (1.76–4.03); null findings after rigorous adjustment	Very serious: directionally inconsistent; IARC OR = 2.2 vs Nordic non-significant OR = 1.4; multiple null findings after confounder adjustment	Downgrade 1 level: substantial I²; effects attenuate toward null with rigorous HPV and behavior adjustment	Downgrade 1 level: directional inconsistency across designs; higher-quality prospective data yield null or near-null results	—
Indirectness	Not serious: direct evidence in WLHIV populations with confirmed HIV status and cervical outcomes	Serious: serology measures historical exposure, not active co-infection; mechanistic evidence predominantly from *in vitro* and non-cervical tissue models	Serious: serology captures lifetime not concurrent exposure; all proposed mechanisms unvalidated in human cervical tissue; no HSV-2 DNA in CC specimens (Tran-Thanh et al., 2003)	Downgrade 1 level: serology as proxy for active infection is indirect; mechanistic human-tissue validation absent	Downgrade 1 level: all three proposed mechanisms (epithelial disruption, immune evasion, hit-and-run) unvalidated in human cervical tissue	—
Imprecision	Not serious: large samples; narrow CIs in primary meta-analyses; RR 2.64 (2.04–3.42) for HPV acquisition; RR ≈6× for CC risk (Stelzle et al., 2021)	Serious: HPV-adjusted independent CT OR = 1.76 (95% CI 1.03–3.01); lower bound approaches clinical null; wide CI range across studies	Serious: Nordic OR = 1.4 (0.9–2.2) crosses unity; most HPV-adjusted estimates non-significant	Contributes to Low–Moderate boundary; HPV-adjusted OR CI spans unity in most rigorous analysis	Reinforces Low: best-quality prospective estimates non-significant	—
Publication bias	Unlikely: large registry-based studies; directional consistency regardless of study size	Suspected: funnel plot asymmetry detected in Naldini et al. (2019); smaller studies report larger effects	Strongly suspected: case–control designs structurally inflate ORs; larger prospective studies non-significant	Downgrade: suspected publication bias inflates pooled OR; Naldini funnel asymmetry documented	Downgrade: strong structural basis for small-study inflation; large studies consistently attenuated	—
Upgrade: dose–response	Yes: continuous inverse CD4–HPV risk gradient; highest risk at CD4 <200 cells/µL (Liu et al., 2018). +1 level upgrade applied	Partial: titre-dependent association in IARC data (Smith et al., 2004); not consistently replicated. No upgrade applied	No: no dose–response demonstrated. No upgrade applicable	N/A	N/A	—
FINAL GRADE RATING	**HIGH**	**LOW–MODERATE**	**LOW**	**Net: 4 downgrade domains; no upgrade. Low–Moderate (not confidently Moderate)**	**Net: 4 downgrade domains; no upgrade. Low**	See above

GRADE ratings: High, confident the true effect is close to estimate; Moderate, moderately confident; Low, limited confidence; Very Low, very uncertain. CT final rating is Low–Moderate because four downgrade domains (risk of bias, inconsistency, indirectness, publication bias) apply, partially offset by a large effect size but not by a dose–response gradient. Statistical significance does not equate to causal certainty. HPV-adjusted estimates should be used preferentially over unadjusted pooled ORs when interpreting clinical significance. CT, *Chlamydia trachomatis*; CC, cervical cancer; CIN, cervical intraepithelial neoplasia; CI, confidence interval; OR, odds ratio; WLHIV, women living with HIV.Bold values in the final row indicate the overall GRADE certainty rating assigned to each co-infection pairing.

Mechanistic studies were included when they: (1) examined a clearly defined pathway relevant to HPV carcinogenesis or STI immune modulation; (2) employed human-derived cell lines or primary human cells preferentially; and (3) were peer-reviewed with clear methodology. Studies were prioritized when independently replicated. All mechanistic evidence is explicitly labeled by evidence level: experimental, correlative, hypothesis-generating, or extrapolative. Human cervical tissue validation status is stated for each claim.

### 2.7 Data synthesis

Primary meta-analysis was not conducted. This decision was pre-specified in the review protocol and reflects five sources of clinical and methodological heterogeneity, detection method, HPV-adjustment status, study design, outcome definition, and geographic/population variation, that would render pooled estimates misleading rather than informative; these are detailed fully in Section 2.8. Pooling effect estimates across these dimensions would produce a summary statistic of indeterminate clinical meaning. This review is therefore conducted as a structured, bias-aware integrative synthesis: evidence is systematically evaluated by domain, effect estimates are reported with study attribution and design context, and GRADE ratings are applied to all key claims (**Table 1**). Narrative synthesis was structured by co-infection type following a consistent framework: (1) epidemiological magnitude, consistency, and heterogeneity; (2) null and contradictory findings; (3) mechanistic plausibility and evidence level; and (4) clinical implications per evidence tier.

To handle overlapping evidence, we used a hierarchy: when both a meta-analysis and its primary studies are discussed, we used only the meta-analysis’s pooled estimate for GRADE certainty, with primary studies included descriptively to show evidence direction. Primary studies are counted separately if they extend population or geographic coverage beyond pooled estimates, e.g., Ferrera et al. (2023) and Ngombe Mouabata et al. (2024). No effect estimate is counted more than once in GRADE ratings. The overlap between meta-analyses and individual primary studies was not verified against each meta-analysis’s included-study list, as noted in Section 10.

### 2.8 Sources of heterogeneity

Five principal sources of between-study heterogeneity are identified across the included literature and are addressed systematically in the results sections for each co-infection pairing.

First, STI detection method is a major driver of heterogeneity. Serology captures historical exposure without confirming active concurrent infection and cannot establish temporal precedence relative to HPV acquisition or CIN development. NAAT-based detection provides evidence of active infection but is sensitive to the timing of sampling. Bhuvanendran Pillai et al. (2022) demonstrated that CT effect estimates attenuate when NAAT-based detection replaces serology, consistent with non-differential misclassification inflating serologically-derived estimates. This source of heterogeneity is expected to elevate I² statistics beyond what would be attributable to true biological variation alone.

Second, HPV adjustment status is the most important source of heterogeneity for the CT and HSV-2 literatures. The progressive attenuation observed in the CT literature, from co-infection OR of 4.03 (95% CI 3.15–5.16) to prospective OR of 2.21 (95% CI 1.88–2.61) to HPV-adjusted independent CT effect of 1.76 (95% CI 1.03–3.01) in Zhu et al. (2016) provides a structured illustration of confounding-driven attenuation. When restricting consideration to HPV-adjusted and prospective study estimates, CT effect sizes attenuate substantially, with the lower confidence bound approaching unity in the most rigorously adjusted analysis. This quasi-sensitivity finding suggests that a meaningful proportion of the observed CT–cervical cancer association is attributable to shared risk factors with HPV rather than to an independent CT biological effect.

Third, study design contributes substantially to between-study variation. Case–control designs are systematically associated with higher effect estimates than prospective cohorts, consistent with selection and recall bias. In the HSV-2 literature, Zhang et al. (2023) explicitly demonstrated that ORs derived from case–control designs exceed those from prospective designs, consistent with retrospective selection bias inflating pooled estimates.

Fourth, geographic variation in co-infection prevalence, sexual behavior patterns, and screening access introduces genuine contextual heterogeneity. CT co-infection rates in HPV-positive women range from approximately 5% in European screening cohorts to 22% in sub-Saharan African settings (Ngombe Mouabata et al. 2024), reflecting true differences in background prevalence and transmission networks.

Fifth, outcome heterogeneity across HPV persistence, CIN2+, CIN3+, and invasive cervical cancer constitutes a form of clinical heterogeneity that cannot be resolved through statistical methods. In the HIV literature, heterogeneity is comparatively lower (I² 40–60% for ART-related outcomes (Kelly et al., 2018a)) partly because HIV status is objectively measured and the CD4 count dose–response provides an anchor that explains a substantial proportion of between-study variation in a mechanistically coherent manner.

### 2.9 Assessment of publication bias and small-study effects

Publication bias was not directly assessable through formal funnel plot construction or Egger’s test, as primary meta-analysis was not performed. Assessment is based on qualitative interpretation of funnel plot asymmetry reported within included meta-analyses and critical appraisal of the structural likelihood of positive-result bias.

For CT–HPV co-infection, Naldini et al. (2019) explicitly reported funnel plot asymmetry across their 48-study meta-analysis, indicating that smaller studies reported disproportionately larger effect estimates. This pattern is consistent with small-study inflation driven by publication bias toward statistically significant positive findings, selective outcome reporting, or both. The funnel plot asymmetry in this meta-analysis warrants caution when interpreting pooled CT co-infection ORs as unbiased estimates of the true association. Zhu et al. (2016) reported no corresponding publication bias analysis, though substantial heterogeneity across their 22 included studies is consistent with small-study effects.

For HSV-2–HPV co-infection, no formal publication bias assessment was reported by Zhang et al. (2023). Given that the majority of the 67 included studies were case–control designs with unadjusted HPV status, and that prospective designs yield materially lower effect estimates in this literature, there is a structural basis for upward bias in the pooled OR of 3.01 (95% CI 2.24–4.04). This estimate should be interpreted as a likely overestimate of any independent HSV-2 effect. The HSV-2 GRADE: low classification reflects, in part, this probable upward bias from unadjusted designs. Visual inspection of the effect estimate distribution suggests small-study effects: the largest and most methodologically rigorous studies (Lehtinen et al., 2002; McClymont et al., 2023) yield the lowest and least significant estimates, consistent with small studies driving the pooled OR upward.

For HIV–HPV co-infection, publication bias risk is substantially lower. Biological plausibility is well established; the dose–response relationship with CD4 count is reproduced across multiple independent cohorts; and the primary meta-analyses draw on large multicenter and registry-based datasets that include attenuated findings alongside positive results. The directional consistency across studies from diverse settings substantially reduces the probability that the HIV–cervical cancer association is an artifact of publication bias. Statistical significance does not equate to causal certainty, but for HIV, the convergence of epidemiological, mechanistic, and dose–response evidence across multiple high-quality sources provides a substantively stronger basis for causal inference than is available for CT or HSV-2.

Overall, publication bias toward positive findings should be considered a probable contributor to upward inflation of CT and HSV-2 point estimates in existing meta-analyses. Users of this evidence synthesis should weight the most rigorously adjusted, prospective, and HPV-status-controlled estimates more heavily than overall pooled meta-analytic ORs when forming clinical judgments.

### 2.10 Causal inference framework

**Table 2** presents a structured Bradford Hill criterion evaluation for each STI–HPV co-infection pairing. These criteria provide a framework for systematic evidence evaluation but cannot establish causality from observational data. The overall causal inference ratings: HIV (strong), CT (probable), HSV-2 (insufficient) reflect composite assessment across all criteria and align with GRADE evidence certainty designations (**Figure 2**).

**Table 2:** Bradford Hill causal inference assessment — structured comparative table.

Bradford Hill criterion	HIV–HPV	CT–HPV	HSV-2–HPV	Basis for rating
Strength of association	Strong: RR≈6× for CC; RR = 2.64 (2.04–3.42) for HPV acquisition (Liu et al., 2018)	Moderate: OR 2.2–4.0 in pooled analyses; attenuates to OR = 1.76 (1.03–3.01) after HPV adjustment (Zhu et al., 2016)	Weak: OR = 2.2 (1.4–3.4) IARC (Smith et al., 2002); RR = 1.0 (0.6-1.7) Nordic (Lehtinen et al., 2002); multiple null findings	Magnitude and consistency of effect estimates across best-available evidence
Consistency	High: confirmed across 38+ studies, multiple continents, diverse designs (Liu et al., 2018; Stelzle et al., 2021)	Moderate: positive in most large observational studies; attenuated or null in studies with rigorous confounder control	Inconsistent: significant in IARC data; non-significant in subsequent cohorts; directionally unreliable	Reproducibility across independent populations and study methodologies
Temporality	Established: HIV precedes HPV acquisition in cohort designs; CD4 decline temporally precedes CIN progression (Liu et al., 2018)	Partial: cross-sectional/case-control dominance prevents temporal sequence confirmation in most studies	Limited: prediagnostic serology cannot establish temporal sequence; cohort data lacking	Availability of longitudinal data establishing exposure before outcome
Dose-response	Confirmed: continuous inverse CD4-HPV risk relationship across studies; risk highest at CD4 <200 cells/µL (Liu et al., 2018)	Partial: titre-dependent association in IARC data (Smith et al., 2004); not consistently replicated	Absent: no dose-response demonstrated in published literature	Biological gradient between exposure intensity and outcome risk
Biological plausibility	Strong: CD4 depletion, CTL impairment well- characterized; HIV *Tat* transactivation plausible (cell model data)	Moderate: LC impairment, NF-κB inflammation, microbiome dysbiosis coherent; limited human validation	Weak: *ICP0*/*ICP4* transactivation in cell models; epithelial disruption plausible; no human cervical tissue validation	Mechanistic coherence with known biology; evidence level of supporting experimental data
Experimental support	Partial: CD4/CTL human immunological data; HIV *Tat* (cell models); ART-mediated risk reduction (Kelly et al., 2018a)	Limited: CT co-culture experiments; microbiome observational data; no human mechanistic RCT	Very limited: cell model transformation; HSV-2 DNA absent from CC specimens; hit-and-run unconfirmed	Quality and translational relevance of experimental evidence
Residual confounding risk	Low-moderate: HIV status objectively measured; dose-response mitigates confounding concern	Moderate-high: sexual behavior is dominant shared risk factor; self-report bias; serology/PCR discordance	High: sexual behavior confounding unresolvable in existing study designs; HSV-2 cross-reactivity bias	Plausibility that observed association reflects unmeasured confounding rather than causal effect
Overall causal inference	Strong — meets most criteria; classified as probable causal co-factor	Probable — most criteria partially met; causal classification requires longitudinal validation	Insufficient — criteria inconsistently met; association does not currently meet causal threshold	Composite assessment across all criteria

Bradford Hill criteria structure causal inference but cannot establish proof. Ratings are evidence-based assessments. Overall causal inference designations (strong/probable/insufficient) align with GRADE levels.

**Figure 2:**
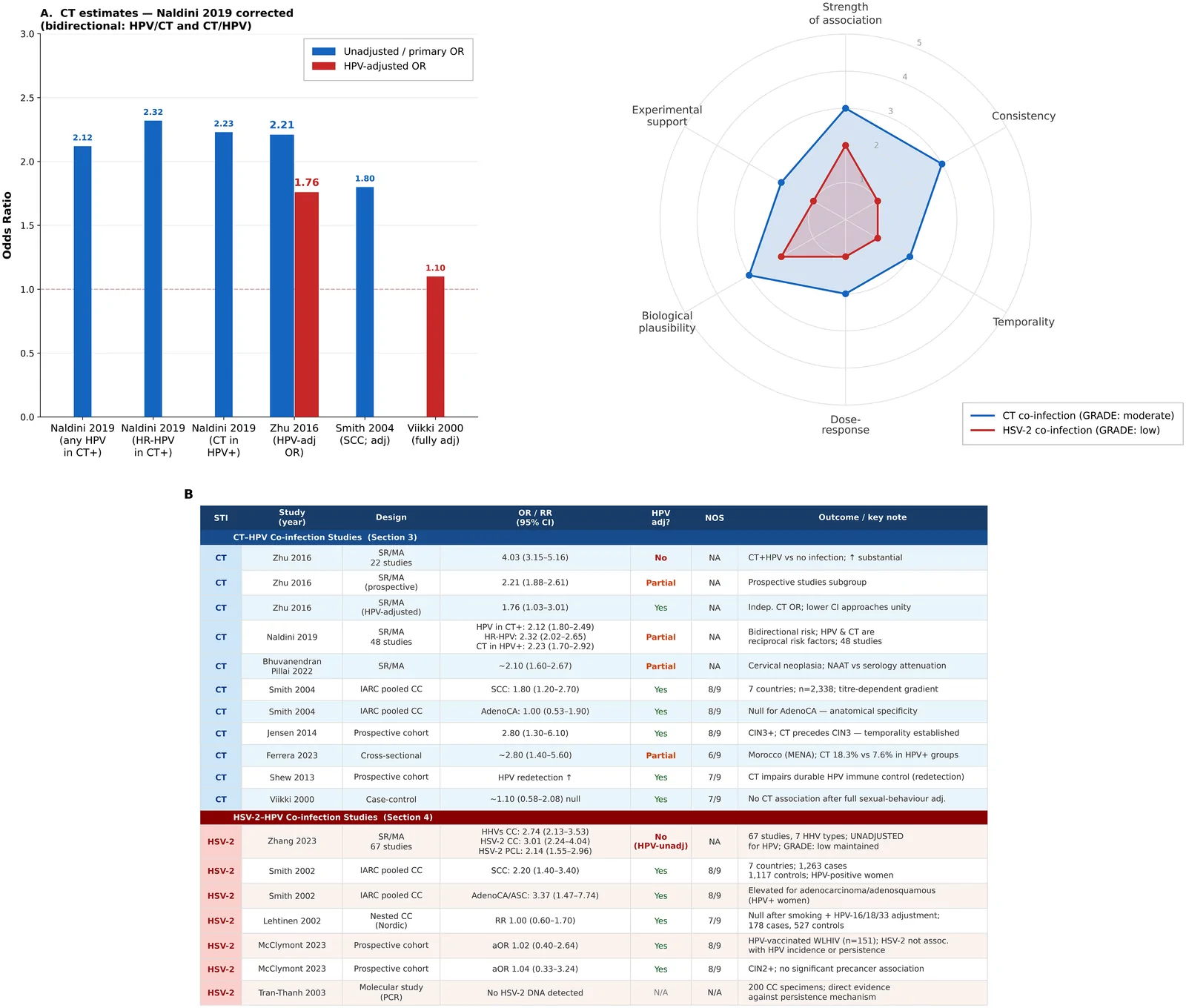
**(A)** CT effect estimates from Naldini et al. (2019)(bidirectional SR/MA), Zhu et al. (2016), Smith et al. (2004), and Viikki et al. (2000). Blue bars = unadjusted/primary OR; red bars = HPV-adjusted OR. Dashed line = null (OR 1.0). The radar chart (right) compares Bradford Hill criteria for CT co-infection (blue; GRADE: Low-Moderate) versus HSV-2 co-infection (red; GRADE: low) across six criteria on a 0-5 scale; CT scores consistently exceed HSV-2 scores across all axes. OR, odds ratio; SR/MA, systematic review/meta-analysis. **(B)** Complete evidence summary for CT-HPV (n = 11 study rows) and HSV-2-HPV (n = 7 study rows) co-infection studies. Columns: study, design, OR/RR (95% CI), HPV adjustment status (green = yes; red = no; orange = partial), NOS score, and outcome note. Zhang et al. (2023) estimates are unadjusted for HPV co-infection status. NOS, Newcastle-Ottawa Scale; OR, odds ratio; RR, relative risk; aOR, adjusted odds ratio; WLHIV, women living with HIV.

As this study is a systematic review of previously published literature, ethical approval and patient consent were not required; no new human or animal data were generated or analyzed.

## 3 HPV and *Chlamydia trachomatis* co-infection

### 3.1 Epidemiological evidence

#### 3.1.1 Prevalence and co-occurrence

CT is the most prevalent bacterial STI globally, with approximately 128 million new infections annually (World Health Organization, 2023b). Among women with confirmed HR-HPV infection in cervical screening settings, CT prevalence ranges from approximately 5% to 20% across diverse geographic contexts (Mosmann et al., 2021; Latorre-Millán et al., 2025). A 2025 prospective cross-sectional study from Spain identified CT as the most frequently detected co-STI in HPV-positive women (5.1% versus 3.8% among HPV-negative women) (Latorre-Millán et al., 2025) (**Table 3**).

**Table 3:** Summary of epidemiological studies — HPV and CT Co-infection.

Author (year)	Country	Design	N	Population	OR (95% CI)	NOS	Key finding / bias note
Zhu et al. (2016)	Global	Meta-analysis (22 studies)	4,291 cases	Sexually active women	CT+HPV OR = 4.03 (3.15–5.16); prospective OR = 2.21 (1.88–2.61)	NA (MA)	I²: significant (substantial heterogeneity); 22 studies predominantly case-control; HPV-adjusted independent CT effect attenuates to OR = 1.76 (1.03–3.01) — lower CI boundary near unity, indicating substantial residual confounding by HPV status.
Naldini et al. (2019)	Global	Systematic review (narrative synthesis) (48 studies)	Multiple cohorts (48 studies)	Sexually active women (48 studies; cohort, CC, cross-sectional)	HPV in CT+ OR = 2.12 (1.80–2.49); HR-HPV OR = 2.32 (2.02–2.65); CT in HPV+ OR = 2.23 (1.70–2.92)	NA (SR)	Funnel plot asymmetry detected: publication bias possible. Case-control dominant. Pooled OR should be interpreted with caution given heterogeneity.
Safaeian et al. (2010)	Costa Rica	Prospective cohort (population-based)	10,049 (182 prevalent + 132 incident CIN2+; 995 controls)	HPV-positive women (HPV-restricted design)	CT DNA: OR = 0.77 (0.42–1.41); CT serology: OR = 1.09 (0.85–1.41) — both null	NR	Large NCI-led cohort with rigorous HPV-restricted design; no association between CT (DNA or serology) and cervical premalignancy after controlling for HPV-positive status.
Smith et al. (2004)	7 countries (IARC)	Pooled case-control	1,238 cases; 1,100 ctrl	HPV-positive women	SCC OR = 1.8 (1.2–2.7); AdenoCA OR = 1.0 (0.53–1.9)	8/9	Association attenuates toward null in some stratified adjustment models; serology only; no adenoCA signal — anatomically consistent but limits scope.
Lu et al. (2024)	China	Cross-sectional immunophenotyping (single-centre)	90 HPV-16-positive women (CT/HPV co-infected vs HPV mono-infected)	Women with HPV-16 infection	Co-infection: reduced Langerhans cell density, CD4+/CD8+ ratio <1, increased Treg infiltration; PI3K activation with MAPK inhibition in Langerhans cells; no lesion-grade outcome reported	7/9	Single-centre; Chinese population; correlative immunological markers in cervical samples, no lesion endpoint — direct validation of the pathway in cervical epithelium remains limited.
Latorre-Millán et al. (2025)	Spain	Cross-sectional (single-centre)	254 HR-HPV+	CC screening women 25–65 y	5.1% CT in HPV+ vs 3.8% HPV−; no lesion outcome reported	8/9	Small; single-centre; cross-sectional co-infection measurement limits temporal inference.
Bhuvanendran Pillai et al. (2022)	Global (SR)	SR & Meta-analysis	Multiple cohorts	Women with CT infection	CT+ elevated cervical cancer odds; effect attenuated with NAAT vs serology	NA (SR)	Confirms CT risk across diverse settings; NAAT-based detection yields lower ORs than serology, highlighting misclassification bias (Bhuvanendran Pillai et al., 2022).
Ji et al. (2019)	China (Inner Mongolia)	Large cross-sectional cohort	>25,000	Women attending cervical screening	CT in 8.4% HPV+ women; CIN2+ significantly elevated vs HPV mono-infection	7/9	Largest Asian cohort; extends evidence to East Asian populations underrepresented in pooled analyses (Ji et al., 2019).
Jensen et al. (2014)	Denmark	Prospective cohort	Registry-based	Women with persistent HR-HPV	CT at baseline: OR = 2.8 (95% CI 1.3–6.1) for CIN3+	8/9	Temporality established: CT precedes CIN3 in longitudinal design; adjusted for HPV genotype and smoking. Strongest causal-inference evidence for CT–CIN3 pathway (Jensen et al., 2014).
Ferrera et al. (2023)	Morocco	Cross-sectional (clinical cohort)	Not specified	HPV+ women, gynecology clinics	CT in 18.3% HPV+ with lesions vs 7.6% HPV+ normal cytology (p<0.05)	6/9	First MENA-region CT/HPV data; small sample, cross-sectional. Addresses geographic representativeness gap (Ferrera et al., 2023).
Ngombe Mouabata et al. (2024)	DR Congo	Cross-sectional	Not specified	HPV+ women, gynecology clinics	CT co-infection 22% in HPV+; elevated HSIL in co-infected vs mono-infected	6/9	Sub-Saharan Africa data; cross-sectional design. Important for LMIC geographic representativeness (Ngombe Mouabata et al., 2024).
Shew et al. (2013)	USA	Prospective cohort	Not specified	Women with apparent HPV clearance	CT associated with HPV redetection after apparent clearance	7/9	Unique temporal evidence: CT may impair durable HPV immune control via redetection mechanism distinct from direct carcinogenesis (Shew et al., 2013).

I² = heterogeneity statistic; substantial = I² > 50%. NOS, Newcastle–Ottawa Scale; MA, meta-analysis; SR, systematic review; CT, Chlamydia trachomatis; HSIL, high-grade squamous intraepithelial lesion; NR, not reported.

#### 3.1.2 Meta-analytic findings and heterogeneity

Zhu et al. (2016) synthesized 22 studies comprising 4,291 cervical cancer cases and reported that CT infection is associated with elevated cervical cancer risk in prospective studies (OR = 2.21, 95% CI 1.88–2.61). HPV/CT co-infection was associated with an OR of 4.03 (95% CI 3.15–5.16) relative to no infection. However, heterogeneity was substantial across included studies (I² significant), and the OR for CT as an independent predictor after HPV adjustment attenuated to 1.76 (95% CI 1.03–3.01), with the lower confidence bound approaching unity. This attenuation illustrates progressive confounder-driven reduction in effect size across studies with more rigorous adjustment (**Figure 3**).

Naldini et al. (2019), in a meta-analysis of 48 studies, reported that among CT-positive women, the OR for any HPV infection was 2.12 (95% CI 1.80–2.49) and for HR-HPV specifically was 2.32 (95% CI 2.02–2.65). Conversely, the OR for CT infection among HPV-positive women was 2.23 (95% CI 1.70–2.92), confirming a bidirectional risk relationship with implications for co-screening strategies. Bhuvanendran Pillai et al. (2022) corroborated this risk elevation in a geographically diverse synthesis, noting that effect estimates were attenuated in studies employing NAAT-based CT detection versus serological methods, highlighting misclassification bias as a source of heterogeneity. Ji et al. (2019) reported CT co-infection in 8.4% of HPV-positive women from Inner Mongolia (n > 25,000), with co-infected women demonstrating significantly higher CIN2+ rates relative to HPV mono-infected women, extending the evidence base to East Asian populations underrepresented in European and North American meta-analyses (**Figures 3**, **4**).

**Figure 3:**
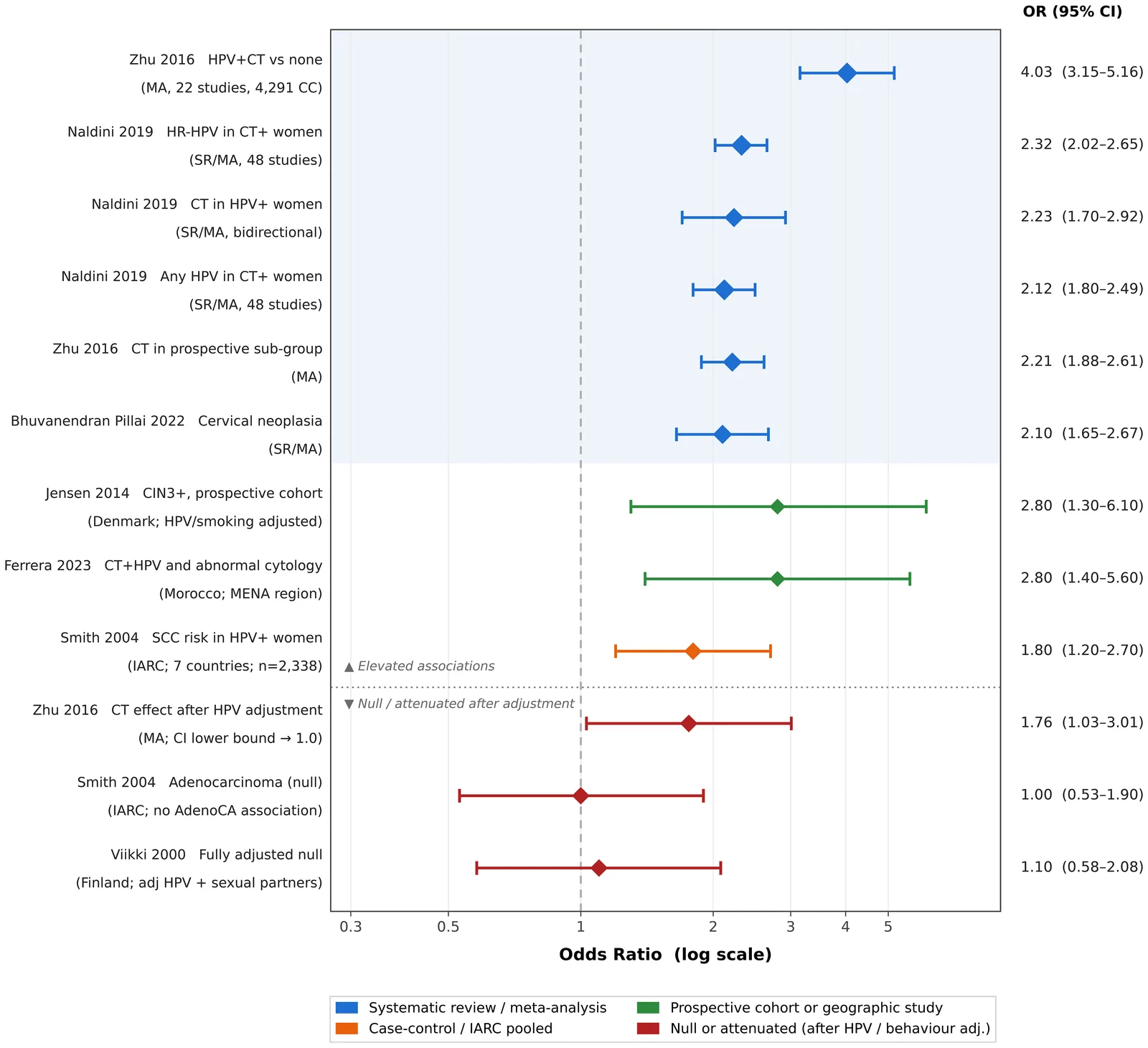
Forest plot of all published CT–HPV co-infection effect estimates for cervical neoplasia outcomes (Section 3). Log scale; box size proportional to study weight; dashed line = null (OR 1.0). Color coding: blue = systematic review/meta-analysis; green = prospective cohort; orange = IARC case-control; red = null or attenuated estimate after HPV/behavior adjustment. The Naldini et al. (2019) meta-analysis presents three bidirectional estimates; the Zhu et al. (2016) HPV-adjusted estimate is shown in red. OR, odds ratio; HR-HPV, high-risk HPV; CC, cervical cancer; MA, meta-analysis.

**Figure 4:**
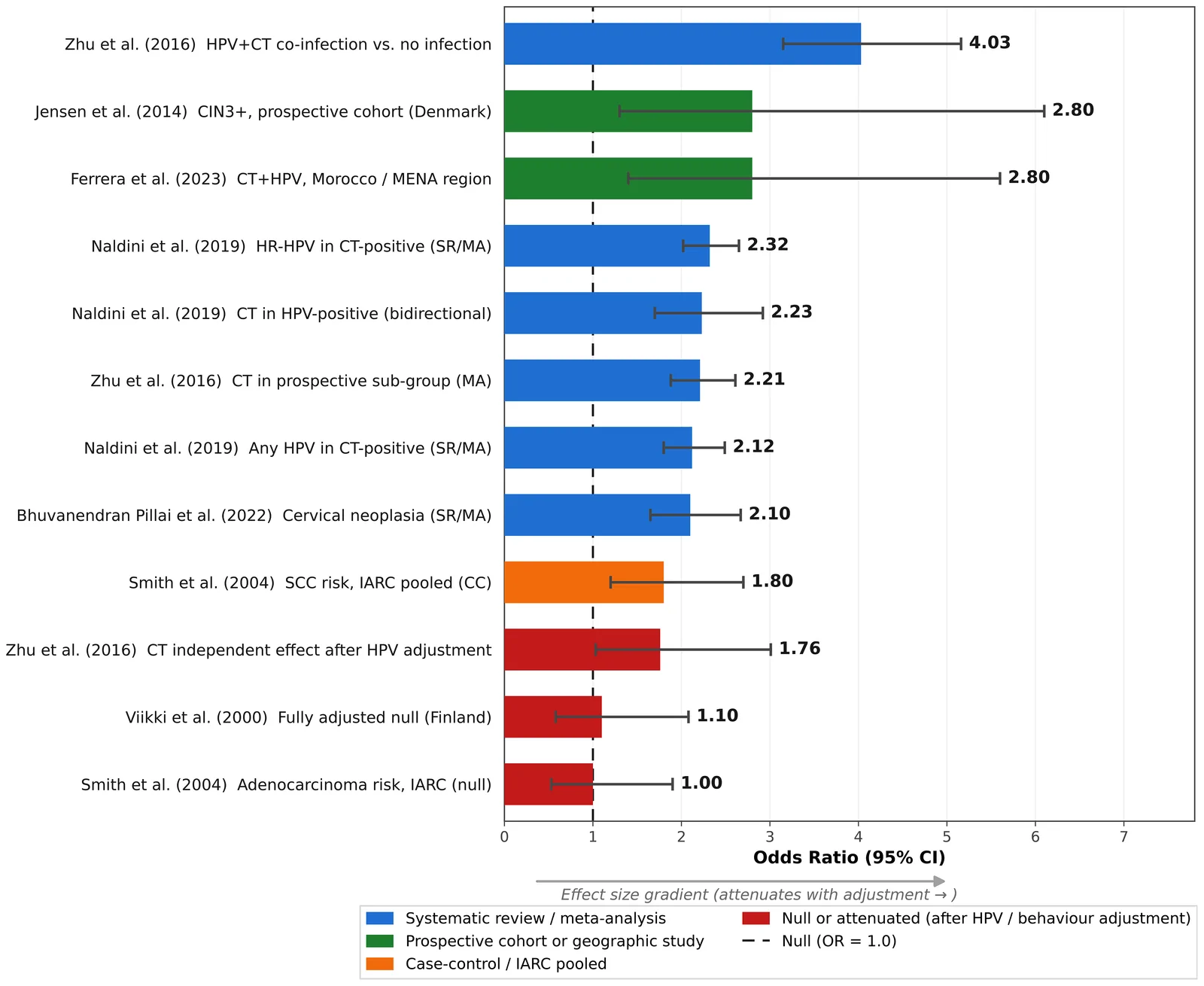
CT–HPV co-infection effect estimates ordered by descending OR magnitude (Section 3). Error bars = 95% CI. The gradient from co-infection OR (4.03) → pooled meta-analytic OR (~2.1–2.3) → IARC-adjusted (1.80) → HPV-adjusted independent CT effect (1.76) → null (1.00–1.10) illustrates progressive confounder-driven attenuation. OR, odds ratio; SCC, squamous cell carcinoma.

#### 3.1.3 IARC pooled case–control evidence

Smith et al. (2004), analyzing 1,238 invasive cervical cancer cases and 1,100 controls from seven countries, reported that CT seropositivity in HPV-positive women is associated with elevated squamous cell carcinoma (SCC) risk (OR = 1.8, 95% CI 1.2–2.7), with a titre-dependent gradient. No corresponding association was observed for adenocarcinoma (OR = 1.0, 95% CI 0.53–1.9), consistent with CT’s selective tropism for squamocolumnar junction cells. This anatomical specificity constitutes one of the more compelling arguments for biological specificity, though it does not establish causality.

#### 3.1.4 Geographic and cohort evidence in under-represented populations

Geographic representation within the CT–HPV co-infection literature remains heavily skewed toward European and East Asian populations. Ferrera et al. (2023) reported a significant association between CT/HPV co-infection and abnormal cervical cytology in a Moroccan clinical cohort, providing rare MENA-region data. CT co-infection was detected in 18.3% of HPV-positive women with cervical lesions compared with 7.6% of HPV-positive women with normal cytology, a difference that persisted after adjustment for age and number of partners, though the study was limited by cross-sectional design and small sample size. Ngombe Mouabata et al. (2024) similarly characterized HPV/CT co-infection in Congolese women, finding 22% CT co-infection rates among HPV-positive women. Jensen et al. (2014) provided important longitudinal evidence from a Danish cohort of women with persistent HR-HPV infection, demonstrating that concurrent CT detection at baseline was associated with elevated CIN3+ risk at follow-up (OR = 2.8, 95% CI 1.3–6.1), even after adjustment for HPV genotype and smoking. The temporality of CT detection preceding CIN3 in this cohort design strengthens causal inference beyond the predominantly cross-sectional and case–control literature.

#### 3.1.5 Contradictory and null findings

Several studies report attenuation or loss of statistical significance after more stringent sexual behavior adjustment. Viikki et al. (2000) found no significant CT association after adjustment for HPV and number of sexual partners. Re-analyses incorporating more comprehensive sexual history data in European cohorts have similarly shown attenuated associations. These null findings raise the substantive possibility that a portion of the observed CT–cancer association reflects residual confounding by sexual behavior rather than an independent CT effect. Conversely, Shew et al. (2013) identified a biologically distinct pathway: CT detection was associated with redetection of HPV after apparent virological clearance, suggesting that CT may impair durable HPV immune control rather than directly promoting carcinogenesis *de novo*. This redetection mechanism, if confirmed in larger cohorts, offers a mechanistically coherent explanation that does not depend solely on direct oncogenic co-operation.

#### 3.1.6 Methodological limitations

Five key constraints limit causal inference. First, case–control dominance prevents establishing temporal sequence. Second, CT serology versus NAAT misclassification: IgG serology reflects historical exposure, not active co-infection, and sensitivity and specificity vary across assays. Third, variable HPV adjustment creates heterogeneity in independent CT effect estimates. Fourth, sexual behavior confounding: lifetime number of partners and condom use are self-reported and subject to recall bias. Fifth, publication bias toward positive findings cannot be excluded. An additional consideration is reverse causality: persistent HPV infection may itself predispose to CT acquisition through cervical microenvironment alterations, consistent with the bidirectional risk pattern reported by Naldini et al. (2019).

### 3.2 Biological mechanisms

#### 3.2.1 Langerhans cell impairment and immune dysregulation

CT infection is associated with impaired Langerhans cell (LC) function in co-culture models and a single-center cohort study. Lu et al. (2024) demonstrated reduced LC density, decreased CD4+/CD8+ T-cell ratio, increased regulatory T-cell infiltration, and PI3K pathway activation with MAPK inhibition in cervical samples from CT/HPV co-infected women (**Figure 5**). Evidence level: correlative, with human cervical tissue data; independent replication required.

**Figure 5:**
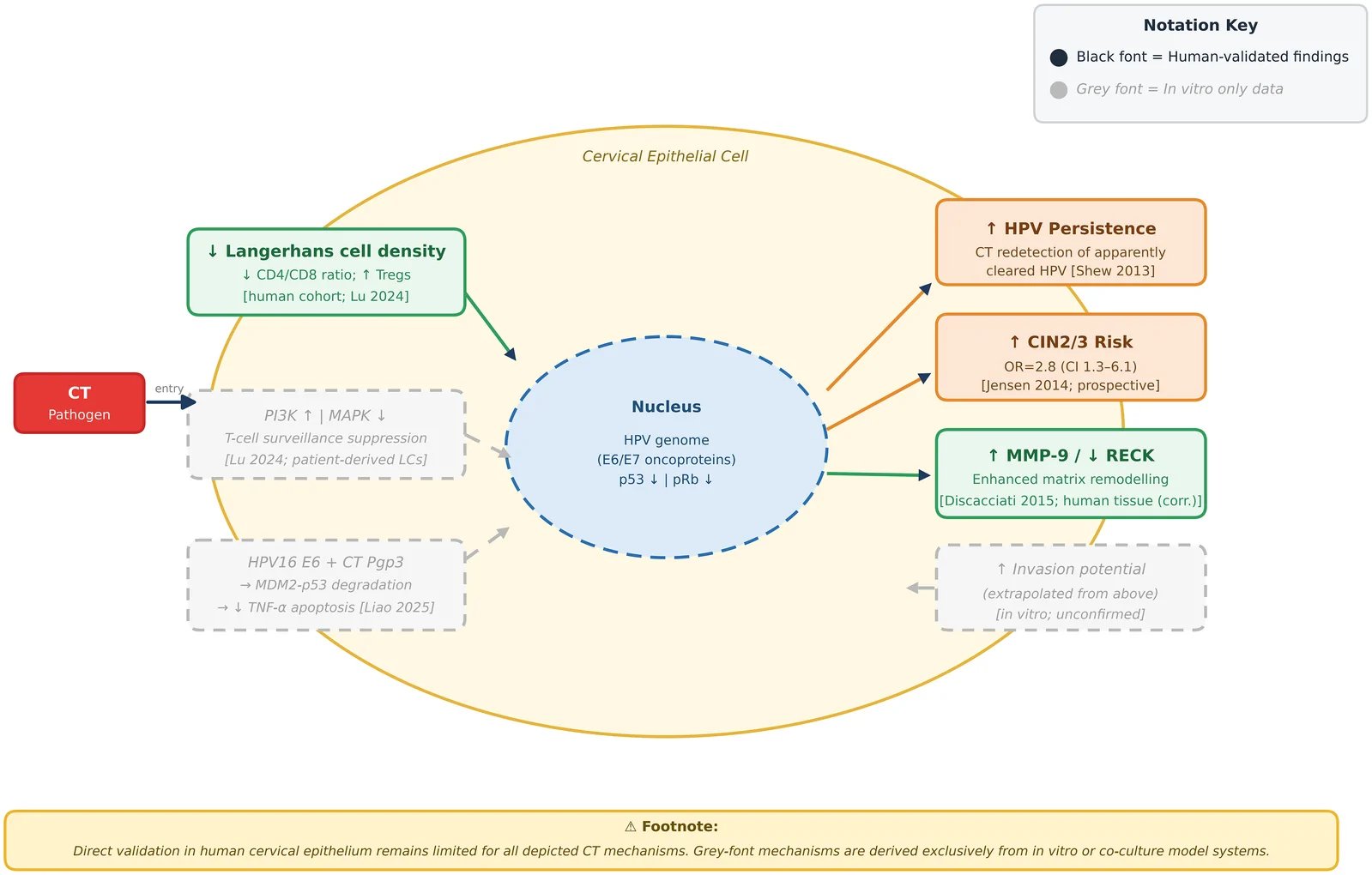
Cell-level mechanistic diagram of CT-HPV interactions within the cervical epithelial cell. Human-validated findings are shown in solid green boxes (black font); in vitro-only mechanisms are shown in gray dashed boxes (gray italic font). Human-validated pathways include decreased Langerhans cell density with altered CD4/CD8 ratio and increased Tregs (Lu et al., 2024), CT-associated HPV redetection supporting increased persistence (Shew et al., 2013), increased CIN2/3 risk (OR 2.8; Jensen et al., 2014), and increased MMP-9/decreased RECK matrix remodeling (Discacciati et al., 2015). In vitro-only mechanisms include PI3K-up/MAPK-down signaling in Langerhans cells with inferred T-cell surveillance suppression (Lu et al., 2024) and HPV16 E6 + CT *Pgp3* MDM2-*p53* degradation with decreased TNF-alpha-induced apoptosis (Liao et al., 2025); these are not validated in human cervical tissue. Direct validation in human cervical epithelium remains limited for all depicted CT mechanisms.

#### 3.2.2 Pro-inflammatory microenvironment and microbiome

Persistent CT infection is associated with upregulation of IL-1β, IL-6, IL-8, and TNF-α through NF-κB activation in *in vitro* systems (Darville and Hiltke, 2010). Whether this inflammatory milieu promotes HPV persistence or accelerates CIN progression in human cervical tissue has not been demonstrated in longitudinal studies; this pathway remains hypothesis-generating (**Figure 5**). CT infection is also associated with reduced *Lactobacillus crispatus* dominance in observational microbiome studies (Leon-Gomez and Romero, 2024); the causal direction of this relationship remains unresolved.

#### 3.2.3 Anti-apoptotic synergism

CT infection has been reported to upregulate anti-apoptotic proteins (Mcl-1, XIAP) in cell culture systems (Darville and Hiltke, 2010), which could theoretically act synergistically with HPV *E6*-mediated *p53* degradation. These data are hypothesis-generating and have not been validated in human cervical tissue. Challagundla et al. (2023) demonstrated in cervical epithelial co-infection models that CT co-operatively enhances HPV *E6*/*E7* expression through shared NF-κB signaling, with elevated PD-L1 expression suggesting concurrent suppression of T-cell surveillance. These findings derive from cell line experiments and require independent replication; their relevance to *in vivo* cervical carcinogenesis in humans cannot be established from this evidence base alone. Liao et al. (2025) characterized a specific protein–protein interaction between HPV16 *E6* and CT outer membrane protein *Pgp3*, demonstrating in cell models that this complex inhibits TNF-α-induced apoptosis through the Daxx-mediated MDM2–*p53* degradation pathway. Discacciati et al. (2015) demonstrated altered MMP-9/RECK ratios in HPV/CT co-infected cervical tissue relative to HPV mono-infection, consistent with enhanced matrix remodeling capacity relevant to CIN progression; this constitutes the most direct human tissue-level mechanistic observation for CT. Evidence level: experimental (*in vitro*, not validated in human cervical tissue) for the *Pgp3*, *E7* and NF-kB findings; correlative (human tissue, cross-sectional) for MMP-9/RECK. Epidemiological evidence, not mechanistic data, should drive clinical conclusions regarding CT’s role in cervical carcinogenesis.

### 3.3 Clinical implications

CT treatment per standard STI guidelines (doxycycline or azithromycin) is indicated for reproductive health benefits (Tier A). Concurrent CT and HPV testing is clinically reasonable (Tier B). CT eradication as a specific cervical cancer prevention strategy is biologically plausible but unsupported by randomized trial evidence (Tier C). No evidence currently supports modification of colposcopy referral thresholds for CT/HPV co-infected women.

## 4 HPV and herpes simplex virus type 2 co-infection

### 4.1 Epidemiological evidence

#### 4.1.1 Global context and key studies

HSV-2 seroprevalence is estimated at 13.2% globally among persons aged 15–49 (James et al., 2020). Smith et al. (2002), analyzing IARC data from 7 countries, reported an association between HSV-2 seropositivity and SCC risk among HPV-positive women (OR = 2.2, 95% CI 1.4–3.4); an association of similar or greater magnitude was reported for adenocarcinoma and adenosquamous carcinoma (OR = 3.37, 95% CI 1.47–7.74). These data do not support histological specificity for HSV-2. The association attenuated with more comprehensive sexual behavior adjustment. Lehtinen et al. (2002) found no association after adjustment for smoking and HPV-16/18/33 (RR = 1.0, 95% CI 0.6-1.7) in a Nordic nested case-control study (178 cases, 527 controls).

Zhang et al. (2023), in a comprehensive meta-analysis of 67 studies examining human herpesvirus infections and cervical carcinoma risk, reported pooled ORs of 2.74 (95% CI 2.13–3.53) for overall herpesvirus–cervical cancer association and 3.01 (95% CI 2.24–4.04) for HSV-2 specifically. However, most included estimates were unadjusted for HPV co-infection status, which substantially limits causal inference. The elevated ORs in traditional case–control designs compared with prospective designs are consistent with retrospective selection bias. These methodological limitations, predominance of unadjusted estimates, structural inflation in case–control designs, and attenuation toward the null in prospective sub-analyses, support the GRADE: low certainty assignment for HSV-2 as an independent carcinogenic co-factor.

McClymont et al. (2023) provided prospective cohort data from 151 HPV-vaccinated WLHIV followed for up to 8 years: HSV-2 seropositivity (76.2% at baseline) was not associated with HPV incidence, persistence (aOR = 1.02, 95% CI 0.40-2.64) or precancerous lesions (CIN2+ aOR = 1.04, 95% CI 0.33-3.24) after adjustment for age and CD4 count, whereas anti-HSV medication use was associated with HSIL cytology. These null findings from the most rigorously designed prospective study argue against HSV-2 serostatus as an independent driver of HPV acquisition or neoplastic progression (**Figure 6**; **Table 4**).

**Figure 6:**
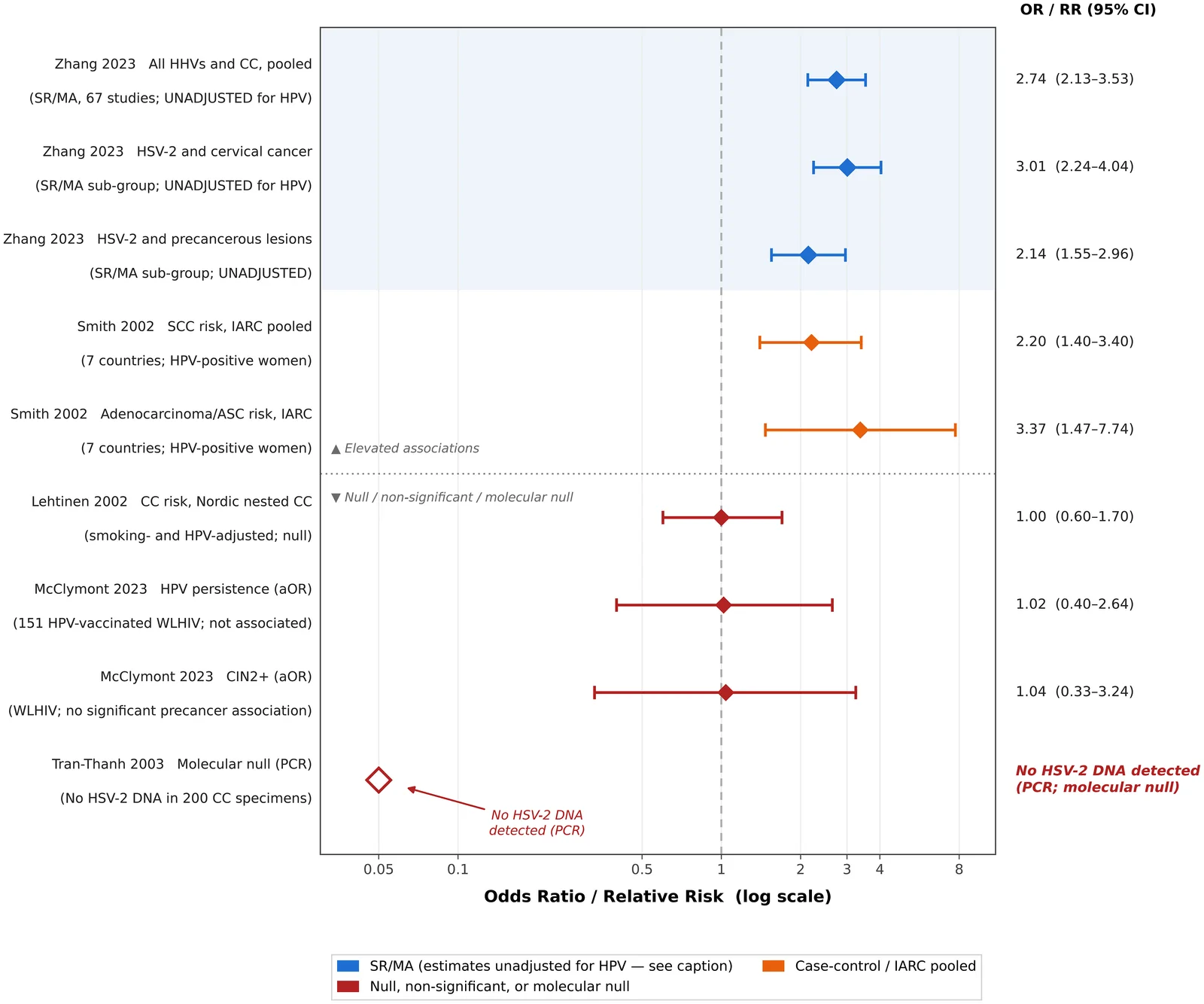
Forest plot of all published HSV-2–HPV co-infection effect estimates for cervical neoplasia outcomes (Section 4). Log scale; box size proportional to study weight; dashed line = null (OR/RR 1.0). Color coding: blue = SR/MA; orange = IARC case-control; red = null, non-significant or molecular null. Tran-Thanh et al. (2003) is represented as an open diamond: no HSV-2 DNA was detected by PCR in 200 cervical cancer specimens. OR, odds ratio; RR, relative risk; aOR, adjusted odds ratio; CC, cervical cancer; PCL, precancerous lesion; WLHIV, women living with HIV.

**Table 4:** Summary of epidemiological studies — HPV and HSV-2 co-infection.

Author (year)	Country	Design	N	Population	OR (95% CI)	NOS	Key finding / limitation
Smith et al. (2002)	7 countries (IARC)	Pooled case-control	1,263 cases; 1,117 ctrl	HPV-positive women	SCC OR = 2.2 (1.4–3.4); AdenoCA/adenosquamous OR = 3.37 (1.47–7.74)	8/9	Association attenuates substantially with comprehensive sexual behavior adjustment; serology only; HSV-1/HSV-2 antibody cross-reactivity possible in some assays.
Lehtinen et al. (2002)	Nordic	Nested case-control	178 cases; 527 controls	Population-based cohort	RR = 1.0 (95% CI 0.6–1.7) after adjustment for smoking and HPV-16/18/33	7/9	Null; confidence interval crosses unity; prediagnostic serology captures historical exposure only.
Tran-Thanh et al. (2003)	Canada	Molecular study	200 CC specimens	Cervical cancer tissue	No HSV-2 DNA by PCR in any specimen	NA	Direct evidence against HSV-2 persistence mechanism; consistent with hit-and-run hypothesis but does not confirm it.
Akbari et al. (2023)	Systematic review	Narrative review	Multiple studies	Anogenital cancers	Conflicting epidemiological results summarized	NA	ICP0/ICP4 transactivation proposed from cell models only; direct validation in cervical epithelium remains limited.
Zhang et al. (2023)	Global (SR)	SR & Meta-analysis (67 studies)	67 studies; 7 HHV types	Women with herpesvirus infection vs controls	Overall HHVs CC OR = 2.74 (95% CI 2.13–3.53); HSV-2 CC OR = 3.01 (95% CI 2.24–4.04); HSV-2 PCL OR = 2.14 (95% CI 1.55–2.96)	NA (SR)	67 studies, 7 HHV types. HSV-2 shows positive correlation with CC and PCL. Estimates unadjusted for HPV status — limits causal inference. Case-control designs yield higher ORs than prospective designs, consistent with retrospective bias. Overall HHV OR driven partly by EBV (OR = 4.89). GRADE: low maintained for HSV-2 as independent co-factor (Zhang et al., 2023).
McClymont et al. (2023)	Multi-country	Prospective cohort (HPV-vaccinated WLHIV)	151	HPV-vaccinated women living with HIV	HSV-2 seropositivity (76.2% at baseline): not associated with HPV incidence, persistence (aOR = 1.02, 95% CI 0.40–2.64) or CIN2+ (aOR = 1.04, 95% CI 0.33–3.24) after adjustment for age and CD4 count	8/9	Prospective design with up to 8 years of follow-up; no association of HSV-2 serostatus with HPV acquisition, persistence or precancer; anti-HSV medication use, not serostatus, was associated with HSIL cytology (McClymont et al., 2023).
Muwonga Tukisadila et al. (2025)	Multi-country	Cross-sectional (female sex workers)	Not specified	Female sex workers; HPV/HIV/HSV-2 syndemic co-infection	Triple co-infection: significantly elevated HPV16/18 viral load vs dual or mono-infection	6/9	Syndemic study: HSV-2 context-dependent effect on HPV replication observed only with concurrent HIV-immunosuppression. Suggests HSV-2 monitoring most relevant in WLHIV rather than immunocompetent women. Cross-sectional; female sex worker population limits generalizability (Muwonga Tukisadila et al., 2025).

Inconsistency across studies and absence of HSV-2 DNA in cervical cancer specimens are primary bases for GRADE: low. No adequate pooled meta-analysis with rigorous confounder control currently available. SCC, squamous cell carcinoma; aOR, adjusted odds ratio; RR, relative risk.

#### 4.1.2 Misclassification bias

Three forms of bias affect HSV-2 epidemiological evidence. First, serology captures lifetime exposure without establishing temporality. Second, HSV-1/HSV-2 antibody cross-reactivity in older assays may inflate effect estimates through false-positive HSV-2 classification. Third, serological positivity does not distinguish between frequent reactivating HSV-2 and latent seropositive status, a distinction that may matter if mucosal disruption is the proposed mechanism.

#### 4.1.3 Contradictory and null findings

Multiple cohort analyses have found no significant association between HSV-2 seropositivity and cervical cancer risk after adjustment for HPV and sexual partners. Tran-Thanh et al. (2003) detected no HSV-2 DNA by PCR in 200 cervical cancer specimens, arguing directly against persistent HSV-2 presence as a mechanism. Muwonga Tukisadila et al. (2025), studying female sex workers, reported that triple co-infection (HPV/HIV/HSV-2) was associated with elevated HPV16/18 viral load compared with dual or mono-infection. While not attributable to HSV-2 alone, this provides preliminary evidence that HSV-2 may exert a context-dependent co-promotional effect on HPV replication specifically when HIV-associated immune suppression is additionally present, a hypothesis distinct from HSV-2 acting as an independent carcinogen in immunocompetent women.

### 4.2 Biological mechanisms

#### 4.2.1 Immunological effects

Active HSV-2 infection impairs antigen presentation through *ICP4*7-mediated TAP blockade, modulates NK cell activity, and suppresses type I IFN responses (Orr and Lanier, 2010). Subclinical HSV-2 reactivation occurs on approximately 28% of sampled days in asymptomatic seropositive persons (Wald et al., 2000). However, these immunological perturbations are characterized predominantly in non-cervical experimental systems, and their impact on HPV-specific immunity in cervical mucosa has not been tested in human tissue.

#### 4.2.2 Epithelial disruption

HSV-2 genital ulceration may create epithelial breaches facilitating HPV basal cell access. However, HSV-2 ulceration is episodic rather than sustained, and whether episodic disruption is sufficient to meaningfully facilitate HPV persistence has not been experimentally demonstrated.

#### 4.2.3 Hit-and-run hypothesis

The hit-and-run model proposes that transient HSV-2 infection causes permanent genomic alterations without requiring persistent viral presence. *ICP0* has been shown to transactivate heterologous promoters in cell models (IARC Working Group, 2007; Everett, 2010); however, this has not been validated in human cervical tissue. The hit-and-run hypothesis remains hypothesis-generating and cannot currently inform clinical management (**Figure 7**).

**Figure 7:**
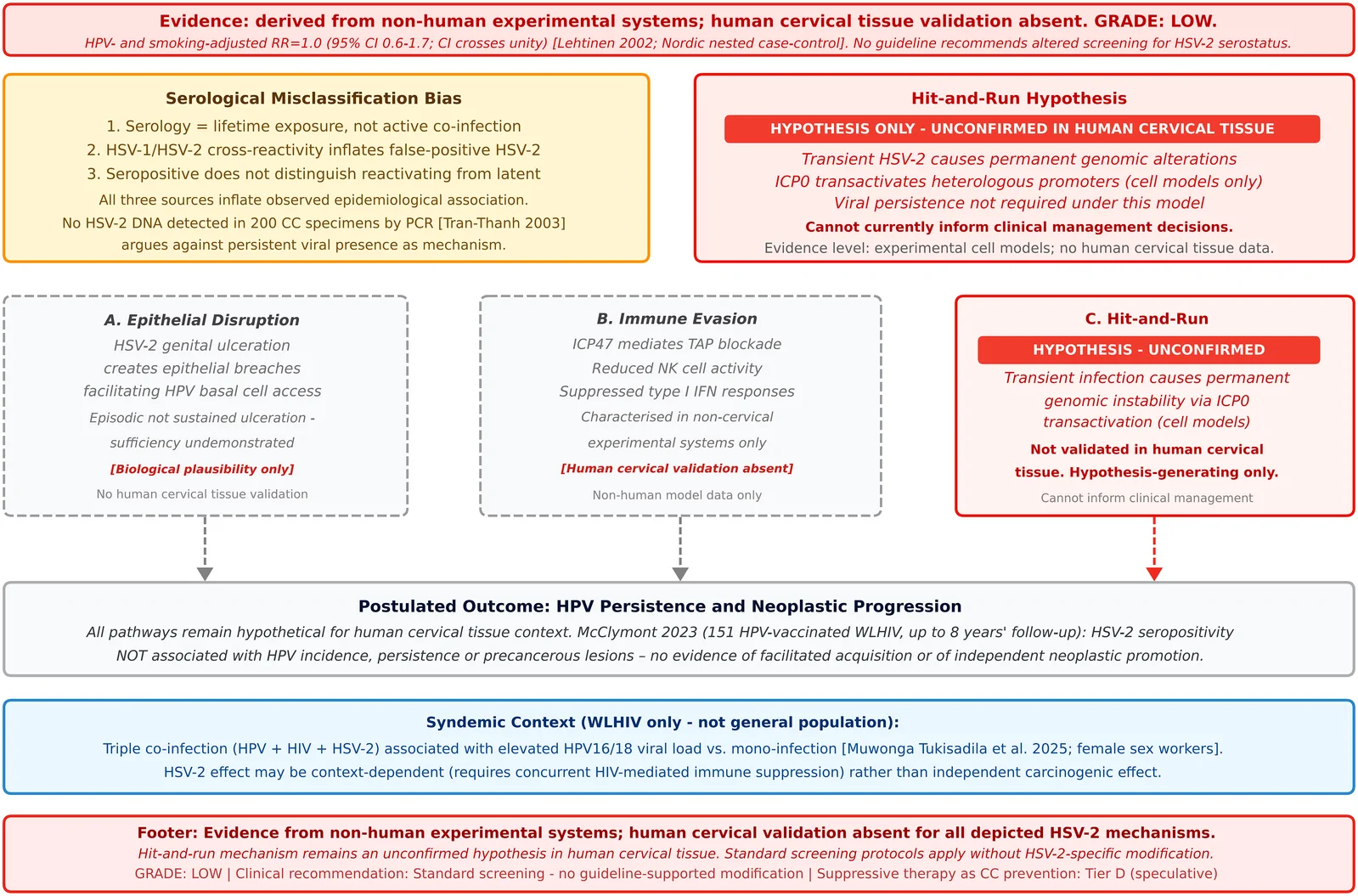
Mechanistic pathways diagram for HSV-2–HPV interactions, corresponding to Sections 4.2.1–4.2.3. The three proposed pathways are shown in dashed boxes to indicate their unvalidated status in human cervical tissue. Serological misclassification biases and the syndemic context are annotated; the effect may be context-dependent, observed only in WLHIV with concurrent immune suppression. GRADE: LOW. Standard cervical screening protocols apply without HSV-2-specific modification.

### 4.3 Clinical implications

Standard cervical screening protocols per WHO and national guidelines apply to women co-infected with HSV-2 without modification. No evidence-based clinical rationale exists for HSV-2 suppressive therapy as a cervical cancer prevention strategy; this is classified as speculative (Tier D).

## 5 HPV and human immunodeficiency virus co-infection

### 5.1 Epidemiological evidence

#### 5.1.1 Global burden and risk magnitude

Cervical cancer is classified as an AIDS-defining illness. WLHIV face approximately 6-fold elevated cervical cancer risk, with approximately 5% of global cervical cancer cases attributable to HIV co-infection; in eastern and southern Africa, this attributable fraction may reach 20–30% (Stelzle et al., 2021; World Health Organization, 2023a).

#### 5.1.2 Antiretroviral therapy and residual risk

Kelly et al. (2018a), in a meta-analysis of 31 ART studies (6,537 WLHIV), reported that ART use is associated with reduced HR-HPV prevalence (aOR = 0.83, 95% CI 0.70–0.99), decreased HSIL-CIN2+ incidence (aOR = 0.59, 95% CI 0.40-0.87), and reduced SIL progression (aHR = 0.64, 95% CI 0.54–0.75). Heterogeneity was moderate (I² = 40–60%), partly attributable to variation in ART regimen, duration, and baseline CD4. Elevated cervical cancer risk persists in ART-treated WLHIV. Kelly et al. (2022), in a separate meta-analysis of 38 studies (18,737 WLHIV, mostly sub-Saharan Africa), reported pooled CIN2+ and CIN3+ prevalence of 12.0% (95% CI 9.8–14.1) and 6.7% (95% CI 5.0–8.4) respectively, with screen-positivity rates varying widely by strategy (VIA 3–31%; HSIL+ cytology 2–46%; HR-HPV DNA 20–64%), underscoring that test choice materially affects over- and under-referral in this population. Swase et al. (2025a), synthesizing 80 studies, confirmed that high-grade lesions persist despite ART, particularly in women with CD4 counts below 350 cells/μL, and that ART adherence and duration are associated with improved outcomes without complete lesion resolution; in the Kelly et al. (2018a) meta-analysis, HR-HPV prevalence was lower among prolonged (≥2 years) than among short-duration ART users (**Table 5**).

**Table 5:** Summary of epidemiological studies — HPV and HIV co-infection.

Author (year)	Region	Design	N	Population	Effect estimate (95% CI)	Quality	Key finding / heterogeneity
Liu et al. (2018)	Global (38 studies)	SR & Meta-analysis	38 studies	HIV+ vs HIV− women	HPV acquisition RR = 2.64 (2.04–3.42); HPV clearance HR = 0.72 (0.62–0.84)	High	I² moderate across outcomes; consistent directional finding in all study designs; dose-response with CD4 strengthens causal inference.
Stelzle et al. (2021)	Global (Lancet GH)	SR & Meta-analysis	Multi-country	WLHIV vs HIV− women	CC risk RR ≈ 6× higher in WLHIV; HIV-attributable fraction ≈ 5% global CC	High	Consistent across regions; highest burden in southern/eastern Africa; heterogeneity partly explained by HIV prevalence and healthcare access.
Kelly et al. (2018a)	Global (Lancet HIV)	SR & Meta-analysis	31 ART studies; 6,537 WLHIV	WLHIV on/off ART	ART: HR-HPV aOR = 0.83 (0.70–0.99); HSIL-CIN2+ incidence aOR = 0.59 (0.40–0.87)	High	I² 40–60%; heterogeneity from ART regimen, duration, baseline CD4; ART reduces but does not eliminate risk.
Cassani et al. (2024)	Global	SR & Meta-analysis	Multiple HIV+ cohorts	WLHIV vs controls	Multiple HR-HPV infections associated with higher HSIL risk	High	Multi-type HPV more common in WLHIV; individual study ORs vary; overall directional consistency.
Pavone et al. (2024)	Review	Review article	WLHIV globally	WLHIV	HIV Tat–HPV E6/E7 transactivation; earlier CC diagnosis in WLHIV	NA	Mechanistic review; experimental data from cell models; direct cervical tissue validation limited.
Swase et al. (2025b)	Global (84 studies)	PRISMA SR	80,023 participants	HPV/HIV co-infected	HSIL prevalent despite ART; HPV-16 dominant; CD4 suppression in subset	High	HSIL risk associated with lower CD4; ART adherence modifies but does not normalize risk.
Swase et al. (2025a)	Global (80 studies)	Systematic review (qualitative synthesis)	80 studies	HPV/HIV co-infected women on ART	No pooled estimate; high-grade lesions persist despite ART, particularly at CD4 <350 cells/μL; ART adherence and duration associated with improved outcomes without complete lesion resolution	High	Residual risk persists despite ART; ART duration and adherence improve but do not normalise outcomes. Supports intensified long-term surveillance regardless of viral load (Swase et al., 2025a).
Olwal et al. (2023)	Computational (Mol Omics)	Network modeling study	Computational dataset	HIV/HPV co-infection molecular pathway analysis	HIV phenocopies PI3K-AKT pathway mutations to enhance HPV-associated cervical cancer signaling	Moderate	Computational convergent mechanism: HIV and HPV may co-operate at PI3K-AKT pathway level. Requires experimental validation. Hypothesis-generating; not clinical evidence (Olwal et al., 2023).
Schindler et al. (2022)	Global (18 studies)	SR (18 studies)	18 studies	WLHIV with and without HR-HPV co-infection	Significantly elevated cervicovaginal IL-6, TNF-α, IP-10 in HIV+/HPV+ vs HIV+ or HPV+ alone; persists on ART	Moderate	Pro-inflammatory cervicovaginal cytokine profile partially persists with virological suppression. Local mucosal inflammation as oncogenic driver beyond systemic CD4 depletion. Human cervical data; correlative (Schindler et al., 2022).
Schindler et al. (2024)	Multi-country	Cross-sectional (HIV-infected women)	Not specified	HIV-infected women; cervicovaginal lavage cytokines vs CIN grade	IL-8 and CXCL10 correlated significantly with HR-HPV load and CIN grade	Moderate	Human cervical tissue-level correlative evidence linking cervicovaginal inflammatory cytokines to neoplastic progression. Cross-sectional; cannot establish causality. Supports residual inflammation hypothesis (Schindler et al., 2024).

I² for ART-related outcomes: 40–60% (Kelly et al., 2018a). All meta-analyses assessed as high quality by GRADE. WLHIV, women living with HIV; ART, antiretroviral therapy; RR, relative risk; aOR, adjusted odds ratio; HSIL, high-grade squamous intraepithelial lesion.

#### 5.1.3 Dose–response and causal inference

Liu et al. (2018), in a meta-analysis of 38 studies, reported elevated HPV acquisition risk (RR = 2.64, 95% CI 2.04-3.42) and reduced clearance (HR = 0.72, 95% CI 0.62-0.84) in women living with HIV, together with a continuous inverse relationship between CD4 cell count and both outcomes, with the lowest clearance below 200 cells/μL.

#### 5.1.4 Multiple HPV genotype co-infections

Cassani et al. (2024) confirmed that multiple concurrent HR-HPV genotype infections, more prevalent in WLHIV, are associated with higher HSIL risk. Swase et al. (2025b), synthesizing 84 studies (80,023 participants), reported that HSIL remains prevalent despite ART with HPV-16 the dominant genotype; the same review demonstrated that WLHIV with CD4 below 350 cells/µL and concurrent HR-HPV showed significantly elevated p16/Ki-67 dual staining positivity compared with WLHIV with CD4 above 500, supporting CD4-stratified screening intensity. Olwal et al. (2023), using network modeling, proposed that HIV infection may functionally mimic PI3K–AKT pathway alterations to enhance HPV-associated cervical cancer signaling. This computational hypothesis requires experimental validation in human cervical tissue before clinical inference can be drawn.

### 5.2 Biological mechanisms

#### 5.2.1 Immunosuppression and HPV-specific immunity

CD4+ T-helper cell depletion reduces HPV-specific cytotoxic T-lymphocyte (CTL) generation, which is essential for immune-mediated clearance of *E6/E7*-expressing keratinocytes (Pavone et al., 2024). This mechanism is directly measurable, dose-dependent, and partially reversible with ART. NK cell dysfunction and impaired antibody-dependent cellular cytotoxicity provide complementary disruptions to immune pathways.

#### 5.2.2 HIV Tat transactivation

HIV Tat has been shown in cell model experiments to transactivate HPV *E6*/*E7* expression through NF-κB signaling and chromatin remodeling at the HPV long control region (LCR) (Pavone et al., 2024), which would amplify *p53* degradation beyond HPV alone. HIV Tat also promotes telomerase activation independently. These data derive from cell models; direct validation in human cervical tissue from WLHIV at defined ART exposures remains a priority. This mechanism is hypothesis-generating and requires human validation before it can inform clinical interpretation.

#### 5.2.3 Residual inflammation despite ART

Residual immune activation, elevated IL-6, D-dimer, and T-cell activation markers persists in many ART-treated WLHIV with sustained virological suppression (Pavone et al., 2024). Schindler et al. (2022), in a systematic review of 18 studies, demonstrated that WLHIV with concurrent HR-HPV show significantly elevated cervicovaginal IL-6, TNF-α, and IP-10 concentrations, with this pro-inflammatory profile partially persisting despite virological suppression on ART. Schindler et al. (2024) further demonstrated that IL-8 and CXCL10 concentrations correlated significantly with HR-HPV load and CIN grade, providing human cervical tissue-level correlative evidence linking local mucosal inflammation to neoplastic progression. Evidence level: correlative human cervical data; causal pathway from residual inflammation to carcinogenesis not established (**Figure 8**).

### 5.3 Clinical course, screening, and prevention

WLHIV present with cervical cancer at a younger age, more advanced stage, higher histological grade, and higher post-treatment recurrence rates (Kelly et al., 2018a). WHO 2021 guidelines recommend initial screening at age 25 with 3-yearly HPV-based testing, and screen-and-treat with VIA in resource-limited settings (Tier A) (World Health Organization, 2021). Two-to-three dose HPV vaccination is required in WLHIV given reduced immunogenicity (Joura et al., 2015). ART optimization constitutes the cornerstone of cervical cancer prevention through immune reconstitution.

In summary, HIV co-infection is associated with approximately 6-fold elevated cervical cancer risk across high-quality meta-analyses, supported by a biologically plausible and partially human-validated immune mechanism with a reproducible dose–response relationship (GRADE: high). The persistence of elevated risk despite ART underscores the need for sustained intensified screening in WLHIV regardless of viral suppression status.

## 6 Triple and multiple STI co-infections

### 6.1 Epidemiology and evidence limitations

Risk factor clustering creates populations in whom simultaneous co-infection with multiple STIs alongside HPV is not uncommon (Patel et al., 2014; Ghosh et al., 2012). However, no dedicated prospective cohort study has directly measured cervical cancer outcomes in women with confirmed triple STI co-infection. All mechanistic and risk discussions in this section represent conceptual extrapolation from individual co-infection data. Akbari et al. (2023) documented HPV co-infection patterns across 87 studies, confirming that CT and HIV are the most frequently documented co-infections in HPV-positive cervical disease, with co-infection rates varying from 5% to 35% by setting. Onyango et al. (2025), in a cross-sectional study of 847 women in Western Kenya using NAAT-based multiplex detection, found that the presence of two or more co-infections was associated with a multiplicative rather than merely additive CIN risk. These findings support biological plausibility for synergistic mechanisms while falling short of confirmation in a dedicated triple co-infection prospective design.

### 6.2 Theoretical synergistic mechanisms

CT-associated LC dysfunction, HSV-2-associated transient IFN suppression, and HIV-associated sustained CD4+ depletion represent three complementary, non-redundant immune pathway disruptions. In combination, these disruptions could plausibly produce additive or super-additive impairment of HPV clearance, potentially through T-cell exhaustion driven by chronic multi-pathogen antigenic stimulation. This pathway remains a mechanistic hypothesis and has not been tested in human cervical tissue or in dedicated triple co-infection cohorts.

### 6.3 Clinical management

Management defaults to individual infection guidelines for each identified pathogen, with multidisciplinary team involvement. No guideline-endorsed protocol exists specifically for multiple STI co-infection. Enhanced surveillance may be appropriate based on cumulative individual risk, but this is extrapolated rather than evidence-based.

## 7 Immunological and molecular cross-talk

### 7.1 Shared immune signaling pathways

Convergent activation of Toll-like receptor (TLR) signaling across STI co-infections may represent a shared oncogenic mechanism. CT activates TLR4; HSV-2 activates TLR2/TLR9; HIV RNA activates TLR7/8 (Kawai and Akira, 2010). This convergence drives NF-κB-mediated inflammatory signaling. A critical distinction must be drawn between acute antiviral TLR activation, which would restrict HPV replication, and chronic low-grade inflammation from persistent co-infection, which may provide a pro-oncogenic microenvironmental advantage. Data on cGAS–STING pathway activation in HPV–STI co-infection at the cervical mucosa are limited and largely extrapolated from non-cervical systems (**Table 6**).

**Table 6:** Biological mechanisms — STI co-infection in cervical carcinogenesis.

Mechanism	CT co-infection	HSV-2 co-infection	HIV co-infection	Evidence level	Key references
Immune suppression	LC impairment; Treg upregulation; CD4/CD8 ratio reduction (in vitro/single-centre cohort; direct cervical epithelium validation limited)	CD4+/CD8+ dysfunction; NK modulation; Treg upregulation; IFN suppression (correlative; non-cervical systems)	CD4+ depletion; CTL dysfunction; well-established in human immunological data; dose-response with CD4 count confirmed	Partial-experimental (CT); Correlative (HSV-2); Well-established (HIV)	Liu et al., 2018; Lu et al., 2024; Pavone et al., 2024
Pro-inflammatory signaling	IL-1β, IL-6, IL-8, TNF-α; NF-κB activation (in vitro; direct cervical epithelium validation limited)	IFN disruption; VEGF upregulation (extrapolated from non-cervical systems)	Persistent IL-6, D-dimer elevation even on ART; T-cell activation markers (human correlative data)	Hypothesis-gen. (CT); Extrapolative (HSV-2); Correlative/human (HIV)	Leon-Gomez and Romero (2024); Pavone et al. (2024)
Oncogene modulation	Anti-apoptotic proteins Mcl-1/XIAP; p53 perturbation (in vitro only; no human cervical tissue validation)	ICP0/ICP4 transactivation (cell models only; NOT confirmed in human tissue; direct cervical epithelium validation absent)	HIV Tat → HPV E6/E7 transactivation (cell model data; plausible; human cervical tissue confirmation needed)	Hypothesis-gen./in vitro only (CT/HSV-2); Experimental — requires human validation (HIV)	IARC Monograph (IARC Working Group, 2007); Pavone et al., 2024
Epithelial disruption	Microabrasion facilitating HPV basal entry (biologically plausible; indirect evidence only)	Mucosal ulceration → HPV basal entry (plausible; transient rather than sustained)	Altered mucosal immunity (plausible; limited direct cervical data)	Plausible — indirect (CT/HSV-2); Plausible — limited direct evidence (HIV)	Smith et al., 2002; Zhu et al., 2016
Microbiome dysbiosis	Lactobacillus reduction; BV dysbiosis (observational human microbiome data; causal direction unconfirmed)	Local ecological disruption (minimally studied; speculative)	BV co-occurrence elevated in WLHIV; cervicovaginal dysbiosis (observational)	Emerging/human observational (CT/HIV); Speculative (HSV-2)	Leon-Gomez and Romero, 2024

*‘Direct validation in cervical epithelium remains limited’ explicitly noted for CT and HSV-2. Evidence levels: Experimental = replicated cell/animal data; Correlative = association without mechanistic proof; Hypothesis-generating = untested in cervical systems; Extrapolative = from non-cervical systems.*

### 7.2 Cervicovaginal microbiome

*Lactobacillus*-dominant cervicovaginal microbiomes, particularly *L. crispatus*, are associated with reduced HPV acquisition and faster clearance in observational studies (Leon-Gomez and Romero, 2024). CT, HIV, and HSV-2 infections are each associated with dysbiotic microbiome shifts in cross-sectional data. Existing predominantly cross-sectional designs cannot establish temporal directionality. Schellekens et al. (2025), synthesizing 34 studies, confirmed that *L. crispatus* depletion and *Gardnerella vaginalis* dominance are consistently associated with HPV persistence and CIN2+ in both HIV-negative and HIV-positive women, and identified that CT co-infection was significantly more prevalent in women with dysbiotic profiles, suggesting a compounding risk pathway. Kongrtay et al. (2025), in a prospective cohort from Kazakhstan, demonstrated that non-vaccinated women with *Lactobacillus*-deficient microbiomes had 2.7-fold higher odds of new HR-HPV acquisition over 12 months, providing longitudinal evidence. Di Pietro et al. (2025), in a systematic umbrella review, characterized a bidirectional CT–microbiome relationship that complicates attribution to a single causal agent. The causal architecture of the CT–microbiome–HPV triad remains unresolved.

### 7.3 Epigenetic modifications

Promoter hypermethylation of tumor suppressors (*CADM1*, *DAPK*, *RARB*) occurs in CIN lesions and may be amplified by chronic inflammation through DNMT1/3 upregulation (Akbari et al., 2023; Steenbergen et al., 2014). The specific contribution of STI co-infection to epigenetic dysregulation in human cervical tissue has not been experimentally characterized and remains hypothesis-generating. The HIV co-infection context provides a partial exception: Kelly et al. (2018b) demonstrated differential *EPB41L3* methylation in cervical tissue from WLHIV compared with HIV-negative women, with methylation levels correlating with CIN grade and HPV16 load. Spatially resolved transcriptomics applied to co-infected cervical tissue represents the highest-priority methodological advance for characterizing these epigenetic alterations at cellular resolution.

### 7.4 Host genetic susceptibility

HLA-DRB1*1301 is associated with reduced cervical cancer risk; cytokine gene polymorphisms modify inflammatory responses and have been associated with variable CIN progression risk (Chen et al., 2013; Mehta et al., 2017). DNA repair gene variants (*XRCC1*, *ERCC2*) may amplify genomic instability; evidence remains preliminary.

## 8 Clinical implications and practice recommendations

All recommendations are explicitly categorized by evidence tier: (A) WHO-guideline-supported; (B) clinically reasonable based on observational evidence; (C) hypothesis-generating; (D) speculative (**Table 7**).

**Table 7:** Clinical practice recommendations by population and evidence tier.

Population	Screening modality	Interval	Recommendations (evidence tier)	Source
General population (no STI)	Cytology or HPV testing (primary HPV preferred)	Every 3–5 yrs from age 25–30	(Tier A) WHO-guideline-supported HPV-based testing	World Health Organization, 2021
HPV+ with CT co-infection	HPV testing + NAAT for CT; standard cytology-based colposcopy threshold	Annual HPV for 2 yrs after CT treatment, then standard intervals	(Tier B) Concurrent HPV/CT testing clinically reasonable. (Tier C) CT treatment as CC prevention: hypothesis-generating, not guideline-supported. Colposcopy threshold modification: not evidence-supported.	Zhu et al., 2016; Naldini et al., 2019; CDC STI Guidelines
HPV+ with HSV-2 co-infection	Standard HPV genotyping and cytology per existing guidelines	Standard interval — no modification for HSV-2 alone	(Tier D) No current guideline recommends altered CC screening thresholds based on HSV-2 serostatus. HSV-2 suppressive therapy as CC prevention: speculative only.	World Health Organization, 2021 — no HSV-2-specific modification; Smith et al., 2002
Women living with HIV (WLHIV)	HPV-based testing preferred; VIA in LMIC per WHO	Every 1–3 yrs from age 25; 3-yearly if 2 consecutive negatives	(Tier A) WHO 2021 guideline: screen from age 25; screen-and-treat in LMIC; 2–3 dose HPV vaccine; ART optimization.	Kelly et al., 2018a; Stelzle et al., 2021; World Health Organization, 2021
Multiple STI co-infections	Standard STI panel; HPV genotyping; standard colposcopy thresholds	Minimum annual; individualized	(Tier C) Multidisciplinary management; no guideline-endorsed protocol for multiple STI co-infection; enhanced surveillance based on clinical judgement.	Cassani et al., 2024; WHO — extrapolated
Resource-limited settings	VIA; self-sampling HPV where available	Single-visit screen-and-treat where feasible	(Tier A) Task-shifting; integrate with HIV care; single-dose HPV vaccination strategy (WHO 2022).	World Health Organization, 2021; Han et al., 2025

Tier A, WHO-guideline-supported; Tier B, clinically reasonable; Tier C, hypothesis-generating; Tier D, speculative. HSV-2 threshold modification: Tier D. CT as CC prevention: Tier C only.

### 8.1 Screening strategies

For WLHIV, WHO 2021 guidelines recommend HPV-based screening from age 25 at 3-yearly intervals (Tier A). In resource-limited settings, VIA screen-and-treat protocols are WHO-endorsed (Tier A) and represent the most pragmatic approach. Self-sampling HPV testing has emerged as an access-expanding strategy (Tier B), with meta-analytic evidence demonstrating comparable sensitivity to clinician-collected samples and improved uptake among hard-to-reach populations (Arbyn et al., 2018). For CT-positive women without HIV, concurrent HPV testing is clinically reasonable (Tier B); standard colposcopy referral thresholds should be maintained. For HSV-2-seropositive women, no guideline modification is warranted (Tier D) (**Table 8**).

**Table 8:** Summary evidence assessment across co-infection pairings.

Co-infection	Epidemiologic strength	Heterogeneity	Mechanistic validation	Confounding risk	GRADE	Clinical confidence / actionability
HPV + HIV	Strong: RR ≈ 6× for CC; RR = 2.64 (2.04–3.42) HPV acquisition; dose-response with CD4 (Liu et al., 2018; Stelzle et al., 2021)	Moderate-high (I² 40–60% for ART studies (Kelly et al., 2018a)); consistent directional finding	CD4+ depletion, CTL impairment well-established in human data; HIV Tat–E6/E7 transactivation in cell models (human cervical validation needed)	Low-moderate; HIV status objectively measured; ART exposure is main residual confounder	HIGH	High: WHO 2021 guideline-endorsed; intensified screening from age 25; multi-dose HPV vaccine; ART as prevention
HPV + CT	Moderate: pooled ORs 2.2–4.0; IARC OR = 1.8 (1.2–2.7) (Smith et al., 2004); attenuates after stringent confounder control; Jensen et al. (2014) cohort OR = 2.8 (1.3–6.1) for CIN3+ adds prospective temporality evidence; Bhuvanendran Pillai et al. (2022) SR and Ji et al. (2019) large Asian cohort extend evidence base; Ferrera et al. (2023) and Ngombe Mouabata et al. (2024) provide first MENA and sub-Saharan Africa data	Substantial (I² significant (Zhu et al., 2016)); OR range wide across designs; bidirectional risk pattern confirmed (Naldini et al., 2019; 48 studies)	LC impairment (in vitro, single-centre cohort); anti-apoptotic effects (in vitro only); direct cervical epithelium validation limited	High: sexual behavior confounding; serology/PCR misclassification; case-control dominance	LOW–MODERATE	Low–Moderate certainty: CT treatment guideline-supported for STI benefit (Tier A); concurrent HPV/CT testing clinically reasonable (Tier B); CT eradication as CC prevention: hypothesis-generating only (Tier C). Causality remains uncertain; avoid clinical overstating of independent CT carcinogenic effect.
HPV + HSV-2	Weak-inconsistent: IARC OR = 2.2 (1.4–3.4) (Smith et al., 2002); null in Nordic cohort RR = 1.0 (0.6–1.7) (Lehtinen et al., 2002); multiple null findings after confounder control	High: directionally inconsistent across studies; no pooled meta-analysis with adequate confounder control available	ICP0/ICP4 transactivation (cell models only); no HSV-2 DNA in CC specimens; hit-and-run unconfirmed in humans; direct cervical epithelium validation absent	High: sexual behavior is dominant confounder; serology captures historical exposure; HSV-1/HSV-2 cross-reactivity possible	LOW	Low: no guideline modification for HSV-2; no screening threshold changes supported; suppressive therapy as CC prevention: speculative
Multiple STIs + HPV	Very limited: no dedicated triple co-infection cohort; conceptual extrapolation from individual pairings only	Unable to assess: absence of systematic data	Purely conceptual: immune exhaustion, T-cell anergy; no experimental evidence in triple co-infection systems	Cannot be assessed	VERY LOW	Very low: defaults to individual infection guidelines; no evidence base for super-threshold clinical interventions

GRADE: HIGH, consistent evidence from well-designed studies with dose–response; MODERATE, mostly consistent with limitations; LOW–MODERATE, evidence base with multiple downgrade domains, where the balance of evidence does not support confident moderate certainty; LOW, inconsistent/limited evidence; VERY LOW, absent adequate evidence. Statistical significance does not equate to causal certainty. Effect estimates cited with study attribution.

### 8.2 Prevention and treatment

HPV vaccination is the cornerstone of primary prevention and should be offered to all eligible individuals regardless of STI status (Tier A). Multi-dose scheduling (2–3 doses) is required for WLHIV owing to reduced immunogenicity (Joura et al., 2015; World Health Organization, 2021) (Tier A). The single-dose HPV vaccination strategy represents a transformative approach for LMICs (Han et al., 2025) (Tier A). CT treatment with doxycycline or azithromycin is guideline-endorsed for STI management (Tier A), though CT eradication as a specific cervical cancer prevention strategy remains hypothesis-generating (Tier C). ART optimization in WLHIV constitutes a fundamental cervical cancer prevention strategy (Tier A). Therapeutic HPV vaccination remains investigational (**Figure 9**).

**Figure 8:**
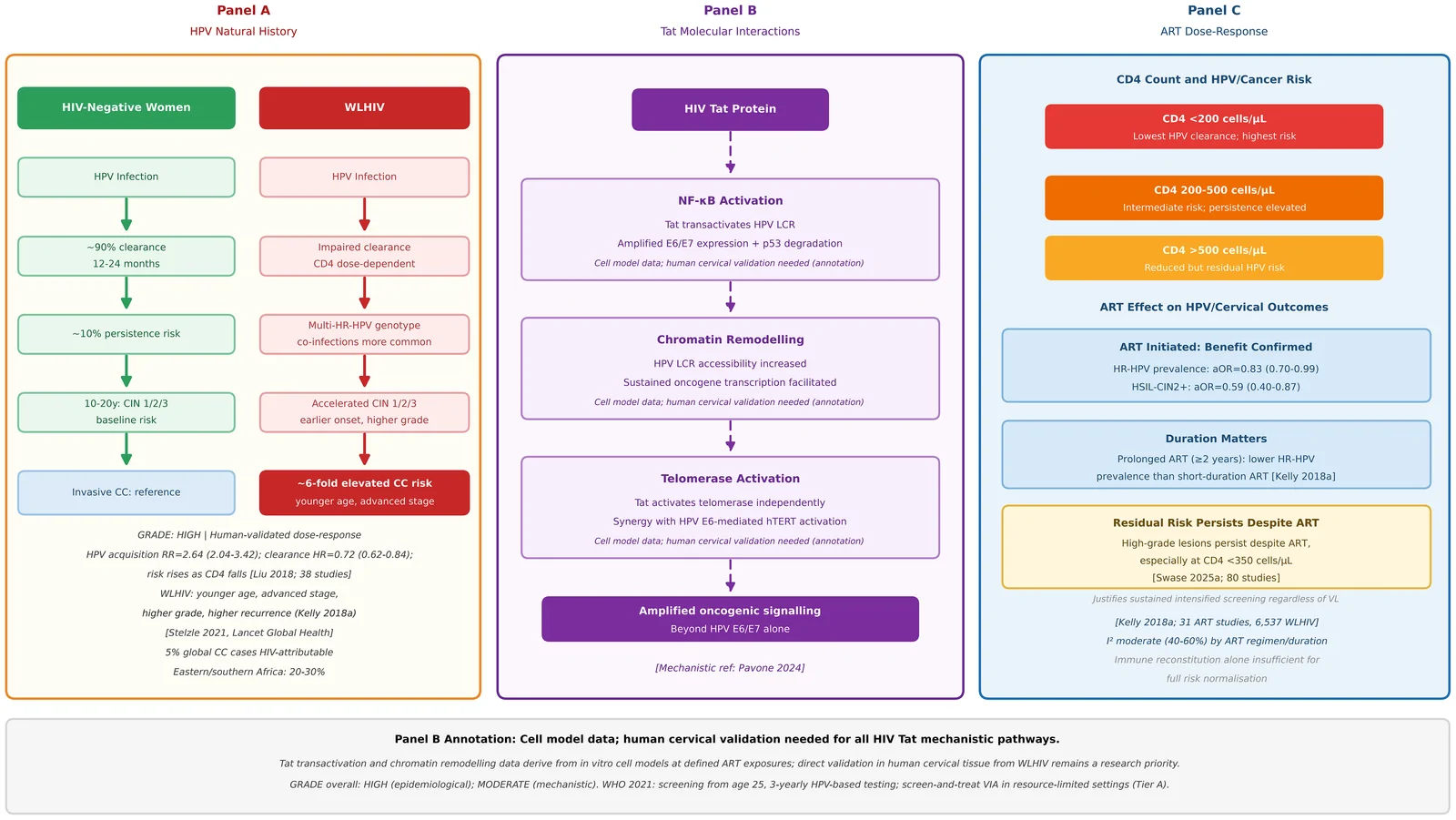
HIV–HPV natural history and molecular mechanisms. Panel **(A)**: Comparative HPV natural history in HIV-negative women versus WLHIV. In HIV-negative women, ~90% of HPV infections clear within 12–24 months, with ~10% persistence risk and 10–20-year CIN 1/2/3 progression baseline. In WLHIV, clearance is impaired in a CD4 dose-dependent manner, multi-HR-HPV genotype co-infections are more common, CIN progression is accelerated with earlier onset and higher grade, and invasive CC risk is approximately six-fold elevated. Panel **(B)**: HIV Tat protein molecular interactions (cell model data; human cervical validation required). Tat activates NF-κB, amplifying HPV LCR-driven *E6*/*E7* expression and *p53* degradation; facilitates chromatin remodeling increasing HPV LCR accessibility; and independently activates telomerase with synergy for HPV *E6*-mediated hTERT activation, producing amplified oncogenic signaling beyond HPV *E6*/*E7* alone. Panel **(C)**: ART dose-response across CD4 count strata. HPV acquisition risk is elevated overall in WLHIV (RR 2.64; 95% CI 2.04-3.42) and rises as CD4 falls, with the lowest clearance below 200 cells/μL; ART reduces HR-HPV prevalence (aOR 0.83) and HSIL-CIN2+ incidence (aOR 0.59), and HR-HPV prevalence is lower with prolonged (≥2 years) than with short-duration ART (Kelly et al., 2018a). High-grade lesions persist despite ART, particularly at CD4 counts below 350 cells/μL (Swase et al., 2025a; 80 studies), justifying intensified screening regardless of viral load. GRADE: HIGH (epidemiological); MODERATE (mechanistic). WLHIV, women living with HIV; ART, antiretroviral therapy; LCR, long control region; CC, cervical cancer; HR-HPV, high-risk HPV.

### 8.3 Resource-limited settings

Single-visit screen-and-treat protocols using VIA followed by immediate cryotherapy or thermal ablation eliminate the two-visit requirement that contributes to losses to follow-up (World Health Organization, 2021) (Tier A). Integration with HIV care platforms is the most efficient delivery model in high-HIV-prevalence settings (Tier A). Task-shifting to trained nurses and community health workers is WHO-endorsed for VIA-based screening (Tier A). Point-of-care HPV testing using platforms such as GeneXpert offers same-day results and higher sensitivity; cost-effectiveness analyses support their use where resources permit (Tier B) (World Health Organization, 2021; World Health Organization, 2023a).

## 9 Knowledge gaps and future research directions

### 9.1 Mechanistic research priorities

The mechanistic understanding of STI co-infection effects on HPV-driven carcinogenesis remains incomplete, with most evidence derived from cell line studies, animal models, or correlative human observations. Human cervical organoid systems and ex vivo tissue explant models represent the most promising validation platforms and should be prioritized (Schutgens and Clevers, 2020). Key unanswered questions include: (1) Does CT infection temporally precede HPV-associated molecular alterations in human cervical tissue, and within what critical window? (2) Does CT eradication reverse Langerhans cell dysfunction and restore HPV-specific immunity, and within what timeframe? (3) Does HIV Tat transactivate the HPV *E6*/*E7* promoter in human cervical tissue at clinically relevant CD4 counts and ART exposures? (4) Do HSV-2 reactivation episodes impair cervical HPV clearance in contemporaneously monitored cohorts, and does suppressive acyclovir modify HPV persistence? Spatially resolved transcriptomics offers particular promise for characterizing co-infection-associated molecular changes at cellular resolution (**Figure 10**).

**Figure 9:**
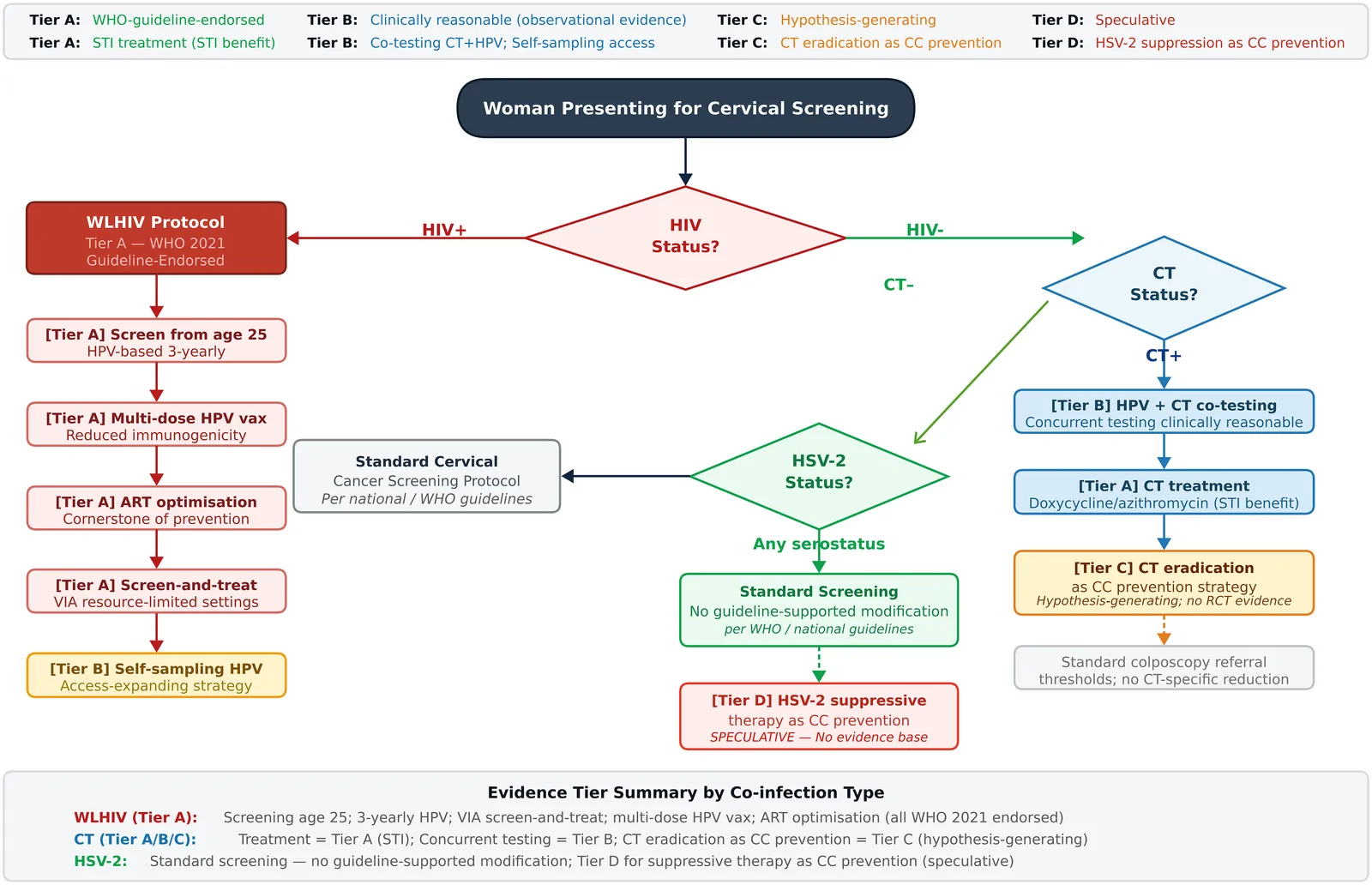
Evidence-tiered integrated cervical cancer prevention algorithm for women presenting for cervical screening, stratified by HIV, CT, and HSV-2 co-infection status. Decision branches are labeled with evidence tiers: Tier A = WHO-guideline-endorsed; Tier B = clinically reasonable based on observational evidence; Tier C = hypothesis-generating, no RCT evidence; Tier D = speculative, no evidence base. HIV-positive women follow the WLHIV protocol (Tier A): HPV-based screening from age 25, multi-dose HPV vaccination, ART optimization, and VIA screen-and-treat in resource-limited settings. CT-positive women in the HIV-negative pathway receive concurrent HPV+CT co-testing (Tier B) and CT treatment with doxycycline/azithromycin (Tier A for STI benefit); CT eradication as a cervical cancer prevention strategy is classified as Tier C (hypothesis-generating; no RCT evidence). HSV-2 serostatus at any level routes to standard screening without guideline-supported modification; HSV-2 suppressive therapy as cervical cancer prevention is classified as Tier D (speculative). VIA, visual inspection with acetic acid; RCT, randomized controlled trial.

### 9.2 Epidemiological research priorities

Large prospective cohort studies with contemporaneous molecular testing for HR-HPV (genotype-specific), CT (NAAT-based), HSV-2 (type-specific serology with viral shedding assessment), HIV (viral load and CD4 monitoring), and cervicovaginal microbiome composition represent the highest priority. Such cohorts should pre-specify HPV clearance, HPV persistence, CIN2+ incidence, and CIN3+/cancer incidence as primary endpoints, and incorporate comprehensive, validated sexual behavior assessment instruments to enable rigorous confounder adjustment. Nested case–control or case–cohort designs within screening programs offer pragmatic alternatives. Geographic data gaps are critical: the Middle East, North Africa, Central Asia, and Pacific regions are substantially underrepresented despite substantial cervical cancer and STI burdens.

### 9.3 Translational and implementation research

Multiplex point-of-care diagnostic platforms capable of simultaneous HPV and co-STI detection are a critical priority, particularly in resource-limited settings. Randomized trials evaluating microbiome restoration strategies with HPV clearance and CIN regression as primary endpoints are needed. Health systems research on co-testing delivery models, patient acceptability, and cost-effectiveness is essential. Implementation science frameworks such as RE-AIM should guide evaluation of integrated screening programs (**Figure 10**).

## 10 Limitations of this review

### 10.1 Protocol registration

The review protocol was developed and followed prospectively but was not formally registered on PROSPERO or an equivalent registry, which limits external verification of the pre-specified methods against a time-stamped public record.

### 10.2 Screening independence

Study selection followed a sequential verification protocol, in which a primary reviewer screened all records and a second author independently verified inclusion decisions and extracted data, with disagreements resolved by consensus. This is not equivalent to fully independent, concurrent dual screening as specified by PRISMA 2020, and inter-rater reliability statistics were not calculated given the sequential design.

### 10.3 Potential overlap between pooled and primary evidence

Several primary studies discussed individually for geographic or population context may also be included within the sample of one or more cited meta-analyses. The hierarchy applied to prevent double counting in GRADE assessment is set out in Section 2.7. The exact degree of overlap was not verified against each meta-analysis’s included-study list, which was beyond the scope of this review. Residual descriptive overlap in the narrative, therefore, cannot be excluded.

### 10.4 Table–figure composition

**Table 3** and the corresponding CT–HPV forest plots (**Figures 2B**, **4**) draw on overlapping but not fully identical study sets: **Table 3** additionally includes Safaeian et al., Lu et al., Ji et al., and Ngombe Mouabata et al. for expanded population and geographic coverage, while the figures additionally include Viikki et al. (2000). Both selections represent the same underlying evidence base; the divergence reflects curation choices made at different stages of the review rather than a substantive discrepancy in findings.

**Figure 10:**
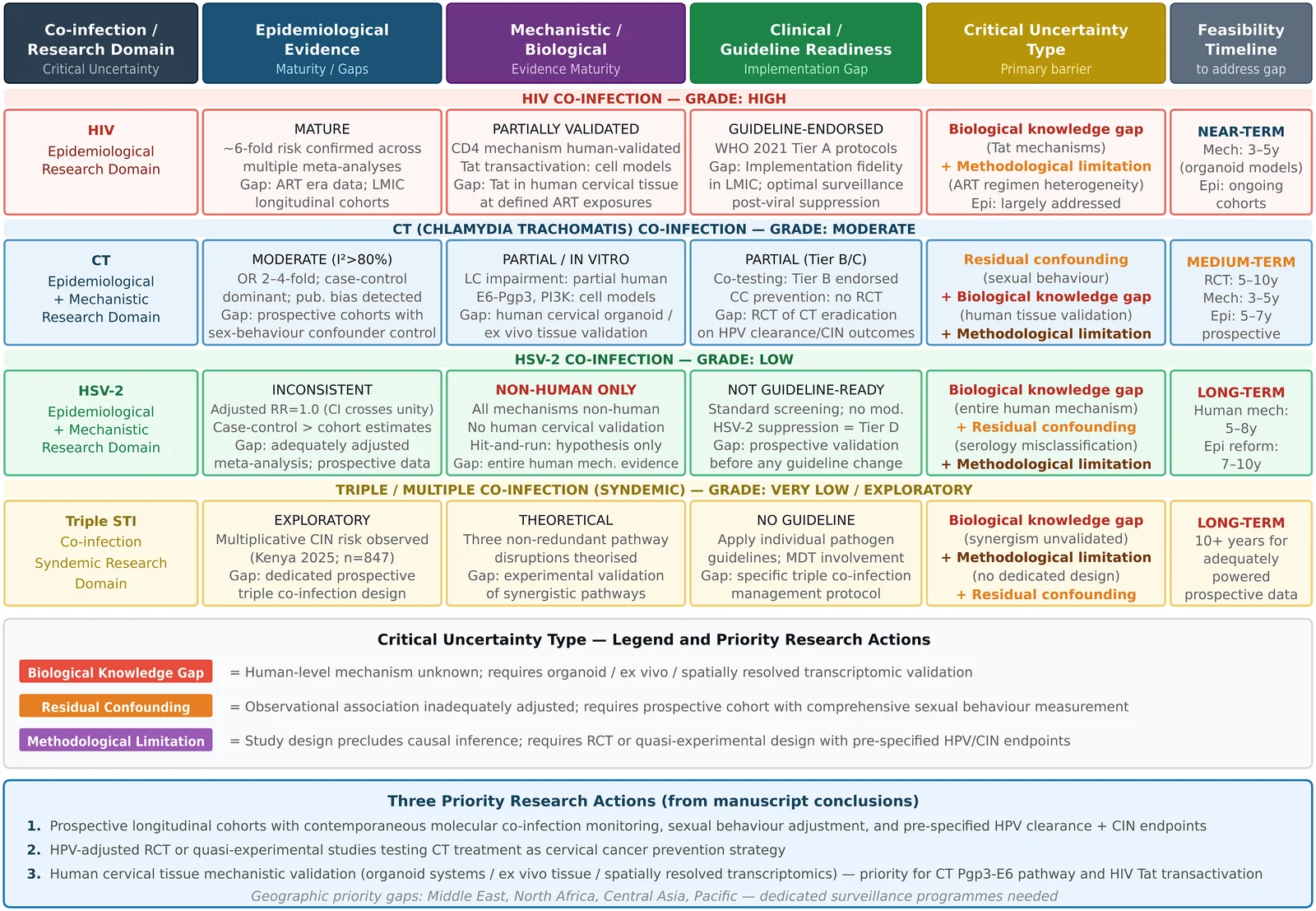
Knowledge gap map across co-infection types, research domains, and evidence maturity. Rows represent four co-infection categories: HIV (GRADE: high), CT (GRADE: moderate), HSV-2 (GRADE: low), and triple/multiple STI syndemic (GRADE: very low/exploratory). Columns characterize each domain across epidemiological evidence maturity, mechanistic/biological validation status, clinical and guideline readiness, primary critical uncertainty type, and feasibility timeline for gap resolution. Critical uncertainty types are color-coded: red = biological knowledge gap (human-level mechanism unknown; requires organoid/ex vivo/spatially resolved transcriptomic validation); orange = residual confounding (observational association inadequately adjusted; requires prospective cohort with comprehensive sexual behavior measurement); purple = methodological limitation (study design precludes causal inference; requires RCT or quasi-experimental design). Three priority research actions identified from manuscript conclusions are listed: (1) prospective longitudinal cohorts with contemporaneous molecular co-infection monitoring and pre-specified HPV clearance and CIN endpoints; (2) HPV-adjusted RCT or quasi-experimental studies testing CT treatment as a cervical cancer prevention strategy; (3) human cervical tissue mechanistic validation using organoid systems or ex vivo tissue with spatially resolved transcriptomics, prioritizing the CT *Pgp3*–E6 pathway and HIV Tat transactivation. Geographic priority gaps: Middle East, North Africa, Central Asia, and Pacific regions. MDT, multidisciplinary team; RCT, randomized controlled trial; LMIC, low- and middle-income countries.

### 10.5 Language and geographic bias

The search was restricted to English-language publications, potentially excluding important studies from the Middle East, North Africa, Central Asia, and other under-represented regions.

### 10.6 Study design constraints

The evidence base is dominated by observational studies (case–control and cross-sectional designs) vulnerable to confounding and bias. Randomized trials are ethically infeasible for STI exposure as a cancer risk factor.

### 10.7 Residual confounding

Sexual behavior is a profound confounder that is difficult to measure accurately. Despite adjustment in included studies, residual confounding cannot be excluded, particularly for CT and HSV-2.

### 10.8 Mechanistic validation gaps

Most mechanistic pathways are supported by *in vitro* or animal model data; human cervical tissue validation is limited for CT and HSV-2.

### 10.9 Detection method heterogeneity

Serological STI detection in many studies reflects lifetime exposure rather than active infection, precluding temporal sequence determination and introducing non-differential misclassification.

## 11 Conclusions

The cumulative evidence supports HIV as a probable biological modifier of HPV-driven cervical carcinogenesis, with high certainty. CT is associated with elevated cervical cancer risk in observational studies, but causal classification remains provisional pending resolution of residual confounding, between-study heterogeneity, and the absence of human cervical tissue mechanistic validation. HSV-2 does not currently meet the evidentiary threshold for independent causal co-factor classification.

For WLHIV, the evidence is most compelling: approximately 6-fold elevated cervical cancer risk, a dose–response relationship with CD4 count, partially validated immune mechanisms, and risk reduction (though not elimination) with ART collectively constitute the strongest available evidence base (GRADE: high). WHO 2021 guideline-endorsed intensified screening from age 25, multi-dose HPV vaccination, and screen-and-treat approaches represent appropriately evidence-grounded clinical standards.

For CT, associated risk elevation in most observational studies is mechanistically coherent but constrained by high residual confounding risk, case–control design dominance, and limited human tissue mechanistic validation (GRADE: Low–Moderate). CT is associated with increased cervical cancer risk in observational data, but causality remains uncertain given residual confounding, substantial heterogeneity, and the attenuation of the HPV-adjusted independent effect toward the null. CT should not be characterized as a confirmed independent co-factor pending prospective validation with rigorous sexual behavior adjustment.

For HSV-2, inconsistent epidemiological findings, absent viral genomic evidence in cancer tissue, and lack of mechanistic validation in human cervical tissue preclude causal classification (GRADE: low). Clinical practice should not be modified based on HSV-2 serostatus alone.

Three research priorities emerge: (1) prospective longitudinal cohort studies with contemporaneous molecular co-infection monitoring, adequate power for sexual behavior adjustment, and pre-specified HPV clearance and CIN progression endpoints; (2) intervention studies testing CT treatment as a cervical cancer prevention strategy in randomized or quasi-experimental designs with HPV-adjusted endpoints; and (3) human cervical tissue mechanistic validation using ex vivo tissue and organoid systems, prioritizing the CT *Pgp3–E6* pathway and HIV Tat transactivation.

The global cervical cancer burden, concentrated in populations with the highest STI co-infection prevalence, makes integration of STI management and cervical cancer prevention programs a coherent public health priority. The precise magnitude of the prevention benefit attributable to broader STI management, however, requires prospective evidence that does not yet exist.

## Data Availability

The original contributions presented in the study are included in the article/supplementary material. Further inquiries can be directed to the corresponding author.
